# Impact of single-room contact precautions on acquisition and transmission of vancomycin-resistant enterococci on haematological and oncological wards, multicentre cohort-study, Germany, January−December 2016

**DOI:** 10.2807/1560-7917.ES.2022.27.2.2001876

**Published:** 2022-01-13

**Authors:** Lena M. Biehl, Paul G. Higgins, Jannik Stemler, Meyke Gilles, Silke Peter, Daniela Dörfel, Wichard Vogel, Winfried V. Kern, Hanna Gölz, Hartmut Bertz, Holger Rohde, Eva-Maria Klupp, Philippe Schafhausen, Jon Salmanton-García, Melanie Stecher, Julia Wille, Blasius Liss, Kyriaki Xanthopoulou, Janine Zweigner, Harald Seifert, Maria J.G.T. Vehreschild

**Affiliations:** 1 Department I of Internal Medicine, Faculty of Medicine and University Hospital of Cologne, University of Cologne, Cologne, Germany; 2 German Centre for Infection Research, partner site Bonn-Cologne, Germany; 3 Institute for Medical Microbiology, Immunology and Hygiene, Faculty of Medicine and University Hospital of Cologne, University of Cologne, Cologne, Germany; 4 Institute of Medical Microbiology and Hygiene, University of Tübingen, Tübingen, Germany; 5 German Centre for Infection Research, partner site Tübingen, Germany; 6 Department of Haematology, Oncology and Immunology, Siloah hospital, Hannover, Germany; 7 Department of Oncology, Haematology, Immunology and Rheumatology, Internal Medicine II, University Hospital Tübingen, Tübingen, Germany; 8 Division of Infectious Diseases, Department of Medicine II, University Medical Centre, Faculty of Medicine, University of Freiburg, Freiburg, Germany; 9 Institute for Medical Microbiology and Hygiene, University Medical Centre, Faculty of Medicine, University of Freiburg, Freiburg, Germany; 10 Department of Haematology, Oncology and Stem Cell Transplantation, University Medical Centre, Faculty of Medicine, University of Freiburg, Freiburg, Germany.; 11 Institute for Medical Microbiology, Virology and Hygiene, University Medical Centre Hamburg-Eppendorf, Germany; 12 German Centre for Infection Research, partner site Hamburg-Lübeck-Borstel, Germany; 13 Department of Oncology and Haematology, Hubertus Wald Tumorzentrum/University Cancer Centre Hamburg, University Medical Centre Hamburg-Eppendorf, Hamburg, Germany; 14 Department I of Internal Medicine, Helios University Hospital Wuppertal, Wuppertal, Germany; 15 Department of Internal medicine I, School of Medicine, Faculty of Health, Witten/Herdecke University, Witten, Germany.; 16 Department of Hospital Hygiene and Infection Control, University Hospital of Cologne, Cologne, Germany; 17 Department of Internal Medicine, Infectious Diseases, University Hospital Frankfurt, Goethe University Frankfurt, Frankfurt am Main, Germany

**Keywords:** infection control, vancomycin-resistant enterococci, single room contact precautions, transmission, immunocompromised host, molecular epidemiology

## Abstract

**Background:**

Evidence supporting the effectiveness of single-room contact precautions (SCP) in preventing in-hospital acquisition of vancomycin-resistant enterococci (haVRE) is limited.

**Aim:**

We assessed the impact of SCP on haVRE and their transmission.

**Methods:**

We conducted a prospective, multicentre cohort study in German haematological/oncological departments during 2016. Two sites performed SCP for VRE patients and two did not (NCP). We defined a 5% haVRE-risk difference as non-inferiority margin, screened patients for VRE, and characterised isolates by whole genome sequencing and core genome MLST (cgMLST). Potential confounders were assessed by competing risk regression analysis.

**Results:**

We included 1,397 patients at NCP and 1,531 patients at SCP sites. Not performing SCP was associated with a significantly higher proportion of haVRE; 12.2% (170/1,397) patients at NCP and 7.4% (113/1,531) patients at SCP sites (relative risk (RR) 1.74; 95% confidence interval (CI): 1.35–2.23). The difference (4.8%) was below the non-inferiority margin. Competing risk regression analysis indicated a stronger impact of antimicrobial exposure (subdistribution hazard ratio (SHR) 7.46; 95% CI: 4.59–12.12) and underlying disease (SHR for acute leukaemia 2.34; 95% CI: 1.46–3.75) on haVRE than NCP (SHR 1.60; 95% CI: 1.14–2.25). Based on cgMLST and patient movement data, we observed 131 patient-to-patient VRE transmissions at NCP and 85 at SCP sites (RR 1.76; 95% CI: 1.33–2.34).

**Conclusions:**

We show a positive impact of SCP on haVRE in a high-risk population, although the observed difference was below the pre-specified non-inferiority margin. Importantly, other factors including antimicrobial exposure seem to be more influential.

## Introduction

The prevalence of colonisation and infection by vancomycin-resistant enterococci (VRE) is increasing globally [1,2]. These gut commensals may cause infections mainly in individuals with immunosuppression, patients being treated at an intensive care unit (ICU), receiving haemodialysis or being extensively exposed to antimicrobials [3].

In order to reduce in-hospital acquisition and transmission of VRE, single-room contact precautions (SCP) including staff and visitors wearing gloves and gowns are recommended by some national infection prevention and control committees [4-6]. However, the underlying evidence is heterogeneous and contradictory [4]. The only two cluster-randomised studies did not observe any impact of targeted or universal wearing of gloves and gowns by staff and visitors on colonisation and infection rates with multidrug-resistant organisms including VRE in ICUs. However, they did not assess the impact of single- vs multiple- bed rooms [7,8]. Similarly, a meta-analysis including eight studies on discontinuation of contact precautions, but not necessarily including single-room accommodation, showed no effect on VRE infection rates [9]. In contrast, a time-series analysis published in 2019, showed a decrease in VRE colonisation and infection rates following a hospital relocation to a building with only single rooms [10]. Furthermore, all available studies lack high-resolution sequence-based typing data to determine transmission of VRE.

Single room contact precautions can have severe effects on patient health including psychological complications, as well as fewer contacts to healthcare providers resulting in a delay in diagnostic and therapeutic procedures [11,12]. Considering the unclear effectiveness and the potential harm associated with SCP, we aimed to assess the impact of SCP on in-hospital acquisition and transmission of VRE in haematological/oncological patients including molecular genetic analyses.

## Methods

### Study design and setting

**Figure 1:**
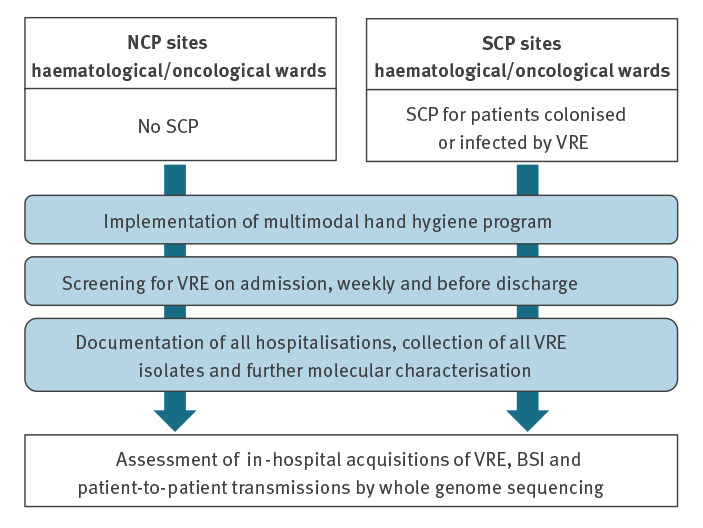
Study design for impact of single-room contact precautions on acquisition and transmission of VRE on haematological and oncological wards, Germany, January −December 2016

BSI: bloodstream infection; NCP: no contact precautions; SCP: single room contact precautions (includes gloves, gowns and single room accommodation); VRE: vancomycin-resistant enterococci.

A prospective 12-month cohort study was performed in 2016 at the haematology/oncology departments of the University hospitals in Cologne, Freiburg, Hamburg and Tübingen. The four sites are situated in different regions of Germany with ca 160 to 700km distance between sites. Inpatients were screened for intestinal colonisation with VRE within 72h of admission, once weekly and within 72h of discharge. As per their standard of care, two sites performed SCP for patients colonised or infected with VRE and two did not (NCP). SCP included accommodation in single rooms with en suite bathrooms and without cohorting, and wearing of gloves and gowns by staff and visitors. Patients at NCP sites and those at SCP sites without known VRE colonisation or infection were accommodated either in single or double rooms. Figure 1 and the Supplementary Table S1 further illustrates design and infrastructure.

To ensure comparability of hand hygiene, sites implemented a multimodal programme including compliance assessment with 150 observations at four different time points per site (Supplementary Text S1, Table S2). The study was registered with clinicaltrials.gov (NCT02623413), the protocol is available at https://www.clinicalsurveys.net/uc/statistic_analysis/1f5d/images/CONTROL_protocol_2015-09_07_MV_signed.pdf .

### Data capture

Inpatients with a minimum stay of one night and at least one screening sample were included. Documentation included demographics, hospitalisation period, and VRE detection. Bloodstream infections (BSI) due to VRE were noted including subsequent hospitalisations within the study period at any ward. Attributable mortality was defined as death within 7 days of onset of VRE BSI or of the last VRE-positive blood culture. Antimicrobial exposure by class was documented until detection of VRE or discharge, with emphasis on antimicrobials previously described as risk factors for VRE acquisition [13-16]. For VRE-positive patients, patient movement data including ward, occupied rooms and occupation dates were documented. Data from multiple hospitalisations in the participating department during the study period (hospitalisation level) were cumulated to patient level. We monitored data completeness and assessed screening adherence, antimicrobial consumption and hand hygiene compliance (Supplementary Text S1).

### Detection of vancomycin-resistant enterococci

Screening for VRE was performed using deep rectal swabs or stool samples plated on selective media (chromID VRE, bioMérieux, Nürtingen, Germany) and incubated for up to 48h. Colonies were identified to species level using mass spectrometry (matrix-assisted laser desorption/ionization time-of-flight, MALDI-TOF), and antimicrobial susceptibility testing was performed using Vitek2 AST P592 cards (bioMérieux) complemented by E-test (bioMérieux) in cases of minimal inhibitory concentrations (MICs) for vancomycin ≥ 4mg/L. In accordance with the European Committee on Antimicrobial Susceptibility Testing (EUCAST) breakpoints, isolates with an MIC value > 4mg/L were considered resistant [17].

### Whole genome sequencing and core genome multilocus sequence typing

The first VRE isolate per patient and BSI isolates were subjected to whole genome sequencing (WGS). Briefly, sequencing libraries were prepared using the Nextera XT library preparation kit (Illumina, Munich, Germany) for a 250 bp paired-end sequencing run on a MiSeq (Illumina). De novo assembly was performed using Velvet (version 1.1.04). Assembled genomes were used for core genome multilocus sequence typing (cgMLST) (1,423 alleles [18],) and traditional 7-loci MLST using SeqSphere + software version 6.0.2 (Ridom, Münster, Germany), van genes were identified using ResFinder (https://cge.cbs.dtu.dk/services/ResFinder/). The raw sequencing reads were submitted to the European Nucleotide Archive under the study accession number PRJEB25579 [19].

### Endpoints and definitions

The primary endpoint was the rate of in-hospital acquisition of VRE (haVRE) in NCP vs SCP sites during 1 year. In-hospital acquisition was defined as a screening or clinical culture obtained > 72h after admission yielding VRE in a patient with a negative admission screening.

In order to test non-inferiority of NCP to SCP in preventing haVRE, a non-inferiority margin of 5% was defined in the statistical analysis plan. Colonisation with VRE does not constitute an immediate hazard to the patient and only few colonised patients develop subsequent infection [20,21]. Based on previously published VRE rates in a similar patient cohort [20], we decided that a difference in the rate of haVRE below 5% would not outweigh the adverse effects of SCP and would not be sufficient to be attributable to SCP.

Secondary endpoints included rates of (i) VRE colonisation overall and at admission, (ii) VRE BSI, and (iii) patient-to-patient transmissions of VRE. Patient-to-patient transmission was defined as the detection of closely related isolates from at least two patients in the same ward with overlapping hospitalisation periods and with at least one patient with haVRE for each transmission event. Close relatedness of isolates was defined as ≤ 10 allele differences in cgMLST, which is lower than the published threshold of 20 alleles to allow for a higher resolution [22]. The term “potential transmission event” was used to refer to pairs or clusters of closely related isolates regardless of confirmation by patient movement data.

Additionally, incidence densities for haVRE and VRE BSI per 1,000 patient days (pd) at risk were calculated counting days at risk from admission to discharge or the day of the respective event (haVRE or VRE BSI). Days of patients already colonised with VRE were censored for haVRE.

### Sample size and statistical analysis

Assuming an overall rate of haVRE of 8% in both groups [20], a one-sided type I error rate of 2.5%, and a dropout rate of 30% due to missed screening, 475 patients per group were required to demonstrate non-inferiority regarding the margin of 5% with a power of 80% (calculated using https://www.sealedenvelope.com/power/binary-noninferior/). To minimise seasonal influence, the study was conducted over 1 year without sample size limitation.

Supplementary Text S3 details the statistical analyses and sensitivity analyses performed. Briefly, distribution of data within groups, both at hospitalisation level (counting each hospitalisation separately) and patient level, was described as absolute numbers plus percentage, mean, median and interquartile range (IQR), and groups compared using Pearson’s chi-squared test, Fisher’s exact test or Mann–Whitney-U tests, as appropriate. Differences in the endpoints comparing NCP to SCP sites were presented as relative risk (RR) with 95% confidence intervals (CI). The effect of covariates and time-varying covariates on haVRE was estimated using the Fine-Gray subdistribution hazard regression model accounting for competing risks [23]. Results were displayed with subdistribution hazard ratio (SHR) and 95% CI. Multiple hospitalisations per patient were included, while hospitalisations of patients already colonised with VRE were excluded. A two-sided p value < 0.05 was considered significant.

### Ethical statement

The study was conducted in line with the Declaration of Helsinki in its revised version of 2013, and approved by the responsible ethical committees (primary vote by Ethics Commission of Cologne University’s Faculty of Medicine – study reference UKK 15–354; further votes by University of Freiburg Ethics Committee; Ethics Commission of the Medical Chamber in Hamburg; Ethics Committee of the Medical Faculty at the Eberhard-Karls University and University hospital Tübingen). Individual consent was waived.

## Results

### Patients and hospitalisations

**Figure 2:**
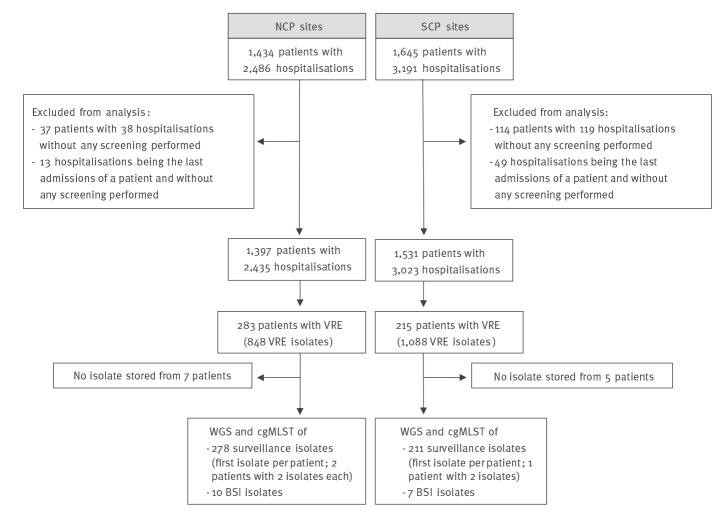
Flow-chart of patient inclusion and isolate collection in multicentre cohort-study on impact of single-room contact precautions on acquisition and transmission of VRE on haematological and oncological wards, Germany, January −December 2016

During 2016, we assessed 1,434 patients corresponding to 2,486 hospitalisations on participating haematological and oncological wards at NCP sites and 1,645 patients corresponding to 3,191 hospitalisations at SCP sites. We included 1,397 patients (2,435 hospitalisations) at NCP and 1,531 patients (3,023 hospitalisations) at SCP sites in the analysis (Figure 2). Table 1 shows patient characteristics. Patients at NCP sites were younger, had a higher proportion of lymphoma as underlying disease and a longer cumulated length of stay. Furthermore, exposure to any antimicrobial was more frequent at NCP sites, while exposure to antimicrobials active against VRE and to fluoroquinolones was more frequent at SCP sites. Findings at hospitalisation level – with each hospital stay of all included patients counted separately – were similar to those at patient level (Supplementary Table S3).

cgMLST: core genome multilocus sequence typing; NCP: no contact precautions; SCP: single-room contact precautions; VRE: vancomycin-resistant enterococci.; WGS: whole genome sequencing.

**Table 1:** Patient characteristics in multicentre cohort-study on impact of single-room contact precautions on acquisition and transmission of VRE on haematological and oncological wards, Germany, January −December 2016 (n= 2,928)

Characteristic	NCP n = 1,397		SCP n = 1,531		p value
	n	%	n	%	
Age in years: Median (IQR)	61 (49–69)	NA	64 (53–75)	NA	< 0.001^a^
Age group					
≤ 40 years	232	16.6	164	10.7	< 0.001^b^
41–60 years	420	30.1	423	27.6	
> 60 years	745	53.3	944	61.7	
Sex					
Female	583	41.7	611	39.9	0.33^c^
Male	814	58.3	920	60.1	
Underlying condition					
Acute leukaemia	225	16.1	288	18.8	< 0.001^b^
Lymphoma	631	45.2	410	26.8	
Solid tumour	356	25.5	465	30.4	
Other	185	13.2	368	24.0	
Patients with multiple hospitalisations	505	36.1	590	38.5	0.19^c^
Hospitalisations per patient: Median (IQR; range)	1 (1–2)	NA	1 (1–2)	NA	0.025^a^
Cumulative length of stay in days: Median (IQR)	15 (7–28)	NA	12 (6–27)	NA	< 0.001^a^
Cumulative length of stay by category					
≤ 6 days	292	20.9	434	28.3	< 0.001**^b^**
7–13 days	349	25.0	371	24.2	
14–27 days	395	28.3	352	23.0	
> 27 days	361	25.8	374	24.4	
Exposure to any antimicrobial class during any hospitalisation	941	67.4	860	56.2	< 0.001^c^
Cumulated exposure to antimicrobials active against VRE (lipopeptides and oxazolidones)^d^					
None	1,353	96.9	1,412	92.2	< 0.001^b^
≤ 7 days	32	2.3	44	2.9	
> 7 days	12	0.9	75	4.9	
Cumulated exposure to cephalosporins^d^					
None	1,239	88.7	1,389	90.7	0.043^b^
≤ 7 days	121	8.7	96	6.3	
> 7 days	37	2.6	46	3.0	
Cumulated exposure to fluoroquinolones^d^					
None	1,221	87.4	1,097	71.7	< 0.001^b^
≤ 7 days	130	9.3	233	15.2	
> 7 days	46	3.3	201	13.1	
Cumulated exposure to glycopeptides^d^					
None	1,265	90.6	1,377	89.9	0.54^b^
≤ 7 days	92	6.6	99	6.5	
> 7 days	40	2.9	55	3.6	
Cumulated exposure to other antimicrobial classes^d^					
None	505	36.1	739	48.3	< 0.001^b^
≤ 7 days	425	30.4	404	26.4	
> 7 days	467	33.4	388	25.3	

IQR: interquartile range; NA: not applicable; NCP: no contact precautions; SCP: single-room contact precautions; VRE: vancomycin-resistant enterococci.

^a^ Mann–Whitney U Test.

^b^ Pearson’s chi-squared test.

^c^ Fisher’s exact test.

^d^ Exposure was assessed for each hospitalisation in days and then cumulated on patient level.

### Vancomycin-resistant enterococci colonisation and bloodstream infections

Overall VRE colonisation or BSI was more frequent at NCP sites with 283/1,397 (20.3%) patients compared with 215/1,531 (14.0%) at SCP sites (RR 1.56; 95% CI: 1.28–1.89). Nearly all VRE isolates were *Enterococcus faecium*, only three patients were colonised with *E. faecalis*. The proportion of patients with haVRE differed significantly between groups with 12.2% (170/1,397) at NCP sites compared with 7.4% (113/1,531) at SCP sites (RR 1.74; 95% CI: 1.35–2.23). This difference of 4.8% was below the pre-specified non-inferiority margin. The corresponding incidence densities of haVRE were 6.93 cases/1,000 pd (95% CI: 5.95–8.04) at NCP and 4.19 cases/1,000 pd (95% CI: 3.47–5.01) at SCP sites. VRE colonisation in admission screenings was slightly more frequent at NCP sites (94/1,397; 6.7%) compared with SCP sites (82/1,531; 5.4%) (RR 1.28; 95% CI: 0.94–1.73).

There were 10 VRE BSIs during the study period at NCP and seven at SCP sites (all *E. faecium*), four of these BSIs in each group occurred during a hospitalisation on a study ward and the remaining six during subsequent hospitalisations on different wards including ICUs. The incidence densities of VRE BSI were 0.33 BSI/1,000 pd (95% CI: 0.17–0.58) at NCP and 0.21 BSI/1,000 pd (95% CI 0.09–0.42) at SCP sites. There was one death attributable to VRE BSI at a SCP site (Supplementary Table S4). The Table 2 shows the rates of VRE endpoints. Median cumulative length of stay until haVRE was 16 days (interquartile range (IQR): 11–38) at NCP and 15 days (IQR: 9–35) at SCP sites (p = 0.475).

**Table 2:** Endpoints for colonisation and BSI in multicentre cohort-study on impact of single-room contact precautions on acquisition and transmission of VRE on haematological and oncological wards, Germany, January −December 2016 (n=2,928)

Outcome	NCP n = 1,397		SCP n = 1,531		RR	95% CI	p value^a^
	n	%	n	%			
haVRE colonisation or BSI	170	12.2	113	7.4	1.74	1.35–2.23	< 0.001
Colonisation with VRE in admission screening	94	6.7	82	5.4	1.28	0.94–1.73	0.12
Overall VRE colonisation or BSI^b^	284	20.3	215	14.0	1.56	1.28–1.89	< 0.001
VRE BSI during hospitalisation on study ward	4	0.3	4	0.3	1.10	0.27–4.39	1.00
VRE BSI^c^	10	0.7	7	0.5	1.57	0.60–4.14	0.47
Close relatedness of VRE isolates by cgMLST^d^	214	15.3	166	10.8	1.41	1.17–1.71	< 0.001
Patient-to-patient transmission of VRE^e^	131	9.4	85	5.6	1.76	1.33–2.34	< 0.001

BSI: bloodstream infection; cgMLST: core genome multilocus sequence typing; CI: confidence interval; haVRE: in-hospital acquisition of vancomycin-resistant enterococci; NCP: no contact precautions; RR: relative risk; SCP: single-room contact precautions; VRE: vancomycin-resistant enterococci.

^a^ Fisher’s exact test.

^b^ One BSI occurred in a patient without prior detected colonisation. Nineteen patients with VRE at NCP and 20 at SCP had no admission screening within 72 hours.

^c^ BSI among study patients at any ward of the participating hospital during study period.

^d^ Number of potential transmission events demonstrated by molecular typing (maximum of 10 allele differences by cgMLST). For example, in a cluster of five isolates there are four potential transmission events.

^e^ Patient-to-patient transmission was defined as close relatedness by cgMLST, overlapping hospitalisation period on same ward and at least one patient per transmission event with haVRE.

### Molecular epidemiology and patient-to-patient transmission

Surveillance isolates were missing in seven NCP and five SCP patients, from two NCP and one SCP patients two isolates each were included due to different resistance patterns. Overall, 287 isolates from 276 NCP patients and 218 isolates from 210 SCP patients were sequenced (Figure 1). *VanB* was the most frequent vancomycin-resistance gene (393/505 isolates; 77.8%) and sequence type (ST)-117 the most frequent MLST type (256/505; 49.8%) followed by ST-80 (91/505; 18.0%) and ST-17 (69/505; 13.7%; Supplementary Table S5, S6).

Using cgMLST, 16 isolate pairs and 28 clonally related transmission clusters involving up to 59 isolates over periods of up to 12 months were detected. We identified 214 and 166 potential transmission events at NCP and SCP sites, respectively (RR 1.41; 95% CI: 1.17–1.71). Integration of patient movement data and information on in-hospital acquisition of VRE confirmed patient-to-patient transmissions in 131/1,397 (9.4%) NCP and in 85/1,531 (5.6%) SCP patients (RR 1.76; 95% CI: 1.33–2.34; Table 2). At NCP sites 72/131 (55.0%) and at SCP sites 55/85 (64.7%) confirmed transmissions were observed between patients sharing the same room. Some patients (7/131 at NCP and 1/85 at SCP sites) with confirmed transmissions had stayed in a room previously occupied by a patient from the same transmission cluster / pair within 7 days. The patient flowchart in Supplementary Figure S1 details the steps of confirmation of patient-to-patient transmission including information on previous room occupancy. Table S7 lists characteristics of detected transmission clusters and isolate pairs, Figures S1a-d show the minimum spanning trees per site.

### Relatedness of bloodstream infections and surveillance isolates

Of 17 VRE BSIs, one occurred in a patient without detected colonisation and in one patient the corresponding surveillance isolate was not available. Of the remaining 15 BSIs, bloodstream isolates were closely related to the corresponding surveillance isolate (Supplementary Figure S3).

### Relatedness of isolates across sites

**Figure 3:**
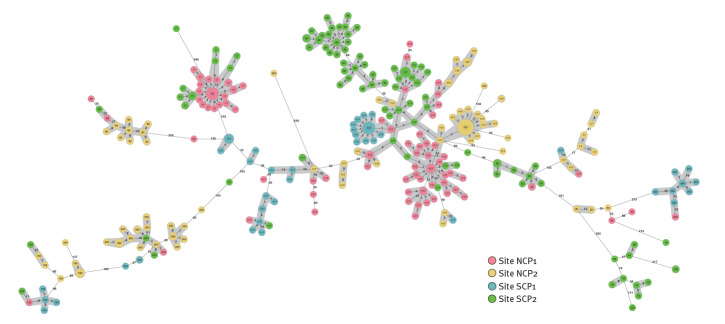
Minimum spanning tree of all VRE isolates from individual patients showing their relatedness as determined by cgMLST, in multicentre cohort-study on impact of single-room contact precautions on acquisition and transmission of VRE on haematological and oncological wards, Germany, January −December 2016

When merging the cgMLST datasets, close relatedness of isolates from different sites became apparent (Figure 3). We identified nine large trans-regional clusters (TRC) comprising up to 99 isolates per cluster from all sites. In total, 297 of 494 sequenced isolates (60.1%) were part of a TRC. ST-117 was the most frequent ST (199/297 isolates; 67.0%). Supplementary Tables S7 and S8 show details of TRC.

cgMLST: core genome multilocus sequence typing; NCP: no contact precautions; SCP: single-room contact precautions; VRE: vancomycin-resistant enterococci.

In this tree, 490 isolates from 490 individual patients are represented. Undivided circles represent one isolate from an individual patient, circles with division lines represent more than one isolate from different patients with 0 allele difference. Labelling of the circles shows sequence type determined by 7-loci sequence typing. Numbers between circles indicate allele differences. A grey connection line or halo shows close relatedness by cgMLST.

### Potential confounders at the patient level

The impact of potential confounders on haVRE was assessed by SHR models accounting for competing risks. In the primary model at patient level, acute leukaemia as underlying disease (SHR 2.34; 95% CI: 1.46–3.75), NCP site (SHR 1.60; 95% CI: 1.14–2.25), the time-varying covariates antimicrobial exposure to cephalosporins (SHR 1.73; 95% CI: 1.26–2.38), fluoroquinolones (SHR 1.61; 95% CI: 1.12–1.96), glycopeptides (SHR 1.61; 95% CI: 1.19–2.18) and other antimicrobial classes (SHR 4.35; 95% CI: 2.84–6.68) were identified as significant risk factors for haVRE (Table 3).

**Table 3:** Risk factors for hospital-acquired VRE colonisation or BSI, in multicentre cohort-study on impact of single-room contact precautions on acquisition and transmission of VRE on haematological and oncological wards, Germany, January −December 2016 (n=2,928)^a^

Variable	Univariate analysis			Multivariate analysis^b^		
	SHR^c^	95% CI	p value	SHR^c^	95% CI	p value
Site group						
SCP	Ref	NA	NA	Ref	NA	NA
NCP	1.71	1.35–2.17	< 0.001	1.60	1.14–2.25	0.007
Age group						
≤ 40 years	Ref	NA	NA	Ref	NA	NA
41–60 years	1.28	0.87–1.88	0.215	1.22	0.82–1.82	0.321
> 60 years	1.27	0.88–1.82	0.199	1.31	0.91–1.91	0.151
Sex						
Female	Ref	NA	NA	Ref	NA	NA
Male	1.22	0.96–1.55	0.112	0.079	0.98–1.58	0.079
Underlying haematological disease in categories						
Solid tumour	Ref	NA	NA	Ref	NA	NA
Acute leukaemia	4.80	3.09–7.46	< 0.001	2.34	1.46–3.75	< 0.001
Lymphoma	2.39	1.55–3.70	< 0.001	1.37	0.87–2.14	0.172
Other	1.25	0.77–2.04	0.368	1.22	0.75–1.99	0.417
Exposure to antimicrobials^d^						
Active against VRE	1.53	0.98–2.37	0.059	0.68	0.42–1.12	0.130
Cephalosporins	3.34	2.55–4.36	< 0.001	1.73	1.26–2.38	0.001
Fluoroquinolones	2.27	1.78–2.90	< 0.001	1.48	1.12–1.96	0.006
Glycopeptides	3.41	2.60–4.48	< 0.001	1.61	1.19–2.18	0.002
Other antimicrobials	6.85	4.66–10.06	< 0.001	4.35	2.84–6.68	< 0.001
Compliance with hand hygiene at respective site during this hospitalisation^d^						
> 75%	Ref	NA	NA	Ref	NA	NA
≤ 75%	1.42	1.13–1.80	0.003	1.03	0.74–1.43	0.883

CI: confidence interval; NA: not applicable; NCP: no contact precautions; Ref: reference; SCP: single-room contact precautions; SHR: subdistribution hazard ratio; VRE: vancomycin-resistant enterococci.

^a^ We considered single- and multiple-record data (multiple hospitalisations) per patient in the model. We excluded patients or single hospitalisations with VRE colonisation at admission. Furthermore, all hospitalisations following a detection of VRE in a prior hospitalisation were excluded. Finally, 2,790 patients with 4,840 hospitalisations were included.

^b^ Adjusted multivariable model for site, age, sex, underlying disease, exposure to different antimicrobial classes, hand hygiene compliance.

^c^ SHR and 95% CI for SHR obtained from the Fine-Gray model.

^d^ Exposure to antimicrobials and compliance with hand hygiene were included as time-varying covariate and could change for each hospitalisation. Exposure to antimicrobials was assessed cumulatively until current hospitalisation, reference category was “no exposure”.

In the sensitivity analyses (Supplementary Tables S9a-i) with different variations of antimicrobial exposure, exposure to any antimicrobial (SHR 7.46; 95% CI: 4.59–12.12; Table S9c) and exposure to antimicrobials not active against VRE (SHR 7.54; 95% CI: 4.64–12.25; Table S9f) showed the strongest association with haVRE and the respective SHRs increased with length of exposure (Tables S9d and S9g). Of note, in these four sensitivity models, hospitalisation at an NCP site was not an independent risk factor for haVRE.

In the analysis at hospitalisation level (Supplementary File 4, Tables S10a-j), acute leukaemia (SHR 1.64–2.84, depending on the model used), NCP site (SHR 1.36–1.87, depending on the model used) and antimicrobial exposure with different variations were, again, independent risk factors. In detail, cephalosporins, glycopeptides and other antimicrobials increased the risk, while antimicrobials active against VRE such as linezolid and daptomycin had a protective effect (Table S10a).

Of note, hand hygiene compliance was not confirmed as an independent risk factor in any of the models.

As an exploratory analysis, we applied the same set of variables to the endpoint confirmed patient-to-patient transmission with similar results (Supplementary Tables S11a-k, S12a-k).

### Potential confounders at study site level

Screening adherence defined as complete admission screening, weekly screenings and one within 72h before discharge was achieved in 70.6% (1,719/2,435) of hospitalisations at NCP and 76.1% (2,299/3,023) at SCP sites. A complete admission screening and one within 7 days before discharge was performed in 88.7% (2,159/2,435) of hospitalisations at NCP and 87.7%% (2,652/3,023) at SCP sites (Supplementary Table S13).

Hand hygiene compliance was assessed less frequently than planned and results differed between sites (Supplementary Figure S4). The compliance at sites NCP2 and SCP1 decreased during the study with compliance as low as 48% at NCP2 during their third and last assessment (Supplementary Figure S3).

Antimicrobial consumption density assessed as defined daily doses per 100 pd differed considerably between sites and groups. SCP sites reported higher consumption of cephalosporins, carbapenems, fluoroquinolones and site SCP1 also of vancomycin. Linezolid and daptomycin consumption varied considerably between sites (Supplementary Tables S14 and S15, Figure S4).

## Discussion

In this large prospective, multicentre study, a significant difference regarding haVRE between NCP and SCP sites was observed. This difference, was below the predefined non-inferiority margin of 5% and thus, according to our estimation, would not outweigh the adverse effects of NCP. We also observed a higher rate of patient-to-patient transmissions determined by cgMLST and patient movement data at NCP sites. Still, with 10 and seven detected BSIs due to VRE these were rare events at NCP and SCP sites. Despite previously reported regional differences in VRE rates in Germany [24], the rate of VRE colonisation at admission differed by only 1.3% between NCP and SCP sites.

The competing risk regression analysis confirmed hospitalisation at a NCP site as an independent risk factor for haVRE in most models. However, exposure to antimicrobials in different variations showed an even stronger and in the sensitivity models more consistent impact on haVRE. In particular, exposures to cephalosporins, fluoroquinolones and glycopeptides were identified as significant risk factors for haVRE as previously reported [15,16,25]. Of note, in the sensitivity models with exposure to any antimicrobial or the group of antimicrobials not active against VRE as risk factors, NCP site was no more significantly associated with haVRE suggesting a less important effect of SCP on the acquisition and/or transmission of VRE as compared with exposure to antimicrobials. Our analysis also revealed an important role of acute leukaemia as underlying disease. This disease is associated with extensive antimicrobial exposure and longer and more frequent hospitalisations compared with other underlying diseases in this cohort, possibly explaining this association.

Interestingly, we identified several TRCs with close relatedness of isolates from different sites. To our knowledge, the present study entails the largest high-resolution sequence-based analysis of trans-regionally collected clinical VRE isolates, but smaller studies have already indicated inter-site relatedness of VRE and in particular the expansion of ST-117 [26,27]. Given the high number of inter-site relatedness and the large geographic distances of participating sites of up to 700 km, direct transfer of individual study patients between sites is not a plausible explanation for this observation. It seems more conceivable that certain VRE clones have a high environmental stability facilitating the observed spread within the healthcare system over long distances and longer time periods than the study period. Since environmental sampling was not included in our study, we unfortunately cannot substantiate this hypothesis further. Still, this assumption, questions our interpretation of direct patient-to-patient transmissions based on genomic relatedness: A certain proportion of the observed transmissions might have been acquisitions from reservoirs in the healthcare environment rather than from patient-to-patient contacts. This highlights the importance of improved environmental cleaning in reducing VRE spread [28]. Looking at details of room occupancy in our study, some transmission might have occurred between patients subsequently but not simultaneously occupying the same room. Further research to investigate the frequency of inter-site relatedness of VRE isolates and trans-regional transmission pathways is needed to determine the clinical relevance of our findings.

Our study has several limitations. Since cluster-randomisation was not feasible due to regulatory reasons, there were clear differences in patient and site characteristics between groups compromising the direct comparison of VRE rates. However, we performed regression models to differentiate the influence of contact precautions from variables with marked variations between groups, e.g. exposure to antimicrobials, underlying disease and hand hygiene compliance. Furthermore, despite rigorous assessment of patient level data and application of up-to-date molecular analysis to investigate epidemiological relatedness of isolates, using only one VRE colony per patient for WGS might have led to underestimation of patient-to-patient transmissions. Since VRE may colonise inanimate surfaces for weeks [29], indirect transmission between patients without simultaneous in-hospital stay may occur but was not assessed. Similarly, we did not record environmental cleaning strategies or perform environmental screening in the participating wards. The existing German recommendations regarding cleaning and disinfection of the hospital environment leave the decision on the frequency of cleaning measures and the exact implementation of quality control to the local hospital hygiene department [30]. Thus, cleaning standards and performance may have varied between the participating sites, influencing the effectiveness of SCP and in-hospital transmission dynamics of VRE. Finally, in a proportion of cases classified as haVRE, VRE might have been present at very low levels in the gut already on admission, only becoming detectable by conventional culture after antimicrobial exposure and successive increase in intestinal bacterial load through antimicrobial selection [31,32].

The findings of this large multicentre study confirm a protective effect of SCP in this specific high-risk population with significant differences in the rates of haVRE and of VRE transmissions. However, estimating the clinical appropriateness of performing SCP remains difficult because of several aspects. Firstly, the observed difference in haVRE rates was just within the predefined non-inferiority margin. We deemed that a difference below 5% would not outweigh the well-known adverse effects of SCP, but surely this estimation is arguable. Secondly, only 0.6% of all patients and 3.4% of VRE colonised patients developed a VRE BSI with only one attributable death in this high-risk population, showing a minor clinical relevance of VRE in our setting [21]. Thirdly, our regression analysis indicates a more relevant and more consistent impact of other factors, particularly antimicrobial exposure, on haVRE. This highlights the importance of antimicrobial stewardship for reduction of VRE burden. Lastly, the role patients colonised with VRE below the cultural detection threshold, as well as inter-site relatedness of isolates and underlying transmission pathways need to be investigated in more detail to understand to which extent the observed relatedness of VRE reflects true transmissions within the hospital that can be prevented by SCP. Though not assessed directly, improved environmental cleaning possibly guided by environmental screening results should be included into infection control strategies for VRE [28].

## Conclusions

The presented evidence suggests a certain protective effect of SCP for haVRE in patients hospitalised on haematological and oncological wards. More importantly, our study adds to the available evidence underlining the exposure to antimicrobials as an important and modifiable factor for the acquisition of VRE.

## Access to data

The raw dataset underlying our analysis is available by contacting the corresponding author upon reasonable request. The raw sequencing reads were submitted to the European Nt Archive (https://www.ebi.ac.uk/ena/), Study Accession number PRJEB25579.

## Supplementary Data

2059000 Supplementary Material
