# Supplementary material for: Impact of single-room contact precautions on acquisition and transmission of vancomycin-resistant enterococci on haematological and oncological wards, multicentre cohort-study, Germany, January−December 2016
Source: Euro Surveill. 2022 Jan 13;27(2):2001876. doi: 10.2807/1560-7917.ES.2022.27.2.2001876 (PMC8759111; doi:10.2807/1560-7917.ES.2022.27.2.2001876)
Supplement: Supplementary Material [file 20-01876_VEHRESCHILD_SupplementaryMaterial.pdf]

Disclaimer: This supplementary material is hosted by Eurosurveillance as supporting information alongside the article ***Impact of single-room contact precautions on acquisition and transmission of vancomycin-resistant enterococci on haematological and oncological wards, multicentre cohort-study, Germany, January-December 2016*** on behalf of the authors who remain responsible for the accuracy and appropriateness of the content. The same standards for ethics, copyright, attributions and permissions as for the article apply. Supplements are not edited by Eurosurveillance and the journal is not responsible for the maintenance of any links or email addresses provided therein.

Supplementary Material

Table of contents

Table S1: Infrastructural information on participating sites and infection control measures 3

Text S1: Description of multimodal hand hygiene program 3

Table S2: Hand Hygiene Programme Modules 4

Text S2: Statistical analysis 4

Table S3: Hospitalisation characteristics and length of stay until VRE acquisition* 6

Table S4: Characteristics of 17 cases with VRE bloodstream infections including attributable mortality 6

Table S5: Distribution of van genes determined by WGS (n=505) 7

Table S6: Distribution of multi-locus sequence types derived from WGS (n=505) 7

Figures S1: Flow chart of stepwise assessment of patient-to-patient transmissions of VRE 9

Figures S2a: Minimum spanning tree of all surveillance isolates showing their relatedness at site NCP1 9

Figures S2b: Minimum spanning tree of all surveillance isolates showing their relatedness at site NCP2 10

Figures S2c: Minimum spanning tree of all surveillance isolates showing their relatedness at site SCP1 11

Figures S2d: Minimum spanning tree of all surveillance isolates showing their relatedness at site SCP2 12

Figure S3: Relatedness of bloodstream and corresponding surveillance isolates from five patients as determined by cgMLST..... 13

Table S7: Details of identified clusters and pairs with close-relatedness among VRE isolates of each site 14

Table S8: Details of multi-site clusters with close-relatedness among VRE from different sites 15

Tables S9a-i: Competing risk analysis for hospital-acquired VRE colonisation or bloodstream infection at the patient level: univariate and multivariate regression – Sensitivity analyses (variations highlighted in yellow) 16

Table S9a: Variation with separate sites (patient level) 16

Table S9b: Variation with exposure to any antimicrobial in days (patient level) 17

Table S9c: Variation with exposure to any antimicrobial irrespective of length (patient level) 18

Table S9d: Variation with length of exposure to any antimicrobials in categories (patient level) 19

Table S9e: Variation with length of exposure to antimicrobials active or not active against VRE in days (patient level) 20

Table S9f: Variation with exposure to antimicrobials active or not active against VRE irrespective of length (patient level) 21

Table S9g: Variation with categorised length of exposure to antimicrobials active or not active against VRE (patient level) 22

Table S9h: Variation with exposure to different antimicrobial groups in days (patient level) 23

Table S9i: Variation with categorised length of exposure to different antimicrobial groups (patient level) 24

Tables S10a-j: Competing risk analysis for hospital-acquired VRE colonisation or bloodstream infection at the hospitalisation level: univariate and multivariate regression – Sensitivity analyses (variations highlighted in yellow) 25

Table S10a: Main model with exposure to different antimicrobial classes (hospitalisation level) 25

Table S10b: Variation with separated sites (hospitalisation level) 26

Table S10c: Variation with length of exposure to any antimicrobial class in days (hospitalisation level) 27

Table S10d: Variation with exposure to any antimicrobial irrespective of length (hospitalisation level) 28

Table S10e: Variation with categorised length of exposure to any antimicrobial (hospitalisation level) 29

Table S10f: Variation with length of exposure to antimicrobials active or not active against VRE in days (hospitalisation level) . 30
Table S10g: Variation with exposure to antimicrobials active or not active against VRE (hospitalisation level) 31
Table S10h: Variation with categorised length of exposure to antimicrobials active or not active against VRE (hosp. level) 32
Table S10i: Variation with length of exposure to antimicrobial class in days (hospitalisation level) 33
Table S10j: Variation with categorised length of antimicrobial classes (hospitalisation level) 34
Tables S11a-k: Competing risk analysis for confirmed patient-to-patient transmission of VRE at the patient level: univariate and multivariate regression – (variations highlighted in yellow) 35
Table S11a: Main model (patient level) 35
Table S11b: Variation with separate sites (patient level) 36
Table S11c: Variation with exposure to any antimicrobial in days (patient level) 37
Table S11d: Variation with exposure to any antimicrobial in days and sites separately (patient level) 38
Table S11e: Variation with exposure to any antimicrobial irrespective of length (patient level) 39
Table S11f: Variation with length of exposure to any antimicrobials in categories (patient level) 40
Table S11g: Variation with length of exposure to antimicrobials active and not active against VRE in days (patient level) 41
Table S11h: Variation with exposure to antimicrobials active and not active against VRE irrespective of length (patient level) ... 42
Table S11i: Variation with categorised length of exposure to antimicrobials active and not active against VRE (patient level) 43
Table S11j: Variation with exposure to different antimicrobial groups in days (patient level) 44
Table S11k: Variation with categorised length of exposure to different antimicrobial groups (patient level) 45
Tables S12a-k: Competing risk analysis for patient-to-patient transmission of at the hospitalisation level: univariate and multivariate regression – Sensitivity analyses (variations highlighted in yellow) 46
Table S121a: Main model with exposure to different antimicrobial classes (hospitalisation level) 46
Table S12b: Variation with separated sites (hospitalisation level) 47
Table S12c: Variation with length of exposure to any antimicrobial class in days (hospitalisation level) 48
Table S12d: Variation with length of exposure to any antimicrobial class in days and separate sites (hospitalisation level) 49
Table S12e: Variation with exposure to any antimicrobial irrespective of length (hospitalisation level) 50
Table S12f: Variation with categorised length of exposure to any antimicrobial (hospitalisation level) 51
Table S12g: Variation with length of exposure to antimicrobials active or not active against VRE in days (hospitalisation level) 52
Table S12h: Variation with exposure to antimicrobials active or not active against VRE (hospitalisation level) 53
Table S12i: Variation with categorised length of antimicrobials active or not active against VRE (hospitalisation level) 54
Table S12j: Variation with length of exposure to antimicrobial class in days (hospitalisation level) 55
Table S12k: Variation with categorised length of antimicrobial classes (hospitalisation level) 56
Table S13: Numbers of hospitalisations with completed surveillance screenings 57
Figure S4: Hand hygiene compliance as determined during observations over the study period 57
Table S14: Defined daily doses of antimicrobial classes per 100 patient days of participating study sites and per quarter 58
Table S15: Average quarterly defined daily doses per 100 patient days of participating sites 58
Figure S5: Antimicrobial consumption in defined daily doses per 100 patient days 59
References 60

Table S1: Infrastructural information on participating sites and infection control measures

Site	NCP1	NCP2	SCP1	SCP2
Is there an Antimicrobial Stewardship program in place?	Yes	Yes	Yes	Yes
Infection control measures				
VRE	No specific contact precautions.		Single rooms, gloves, gowns.	
F3GCR-EC	No specific contact precautions.		Single rooms, gloves, gowns.	
Infection control measures for other bacterial or viral infections	According to KRINKO* guidelines	According to KRINKO* guidelines	According to KRINKO* guidelines	According to KRINKO* guidelines
Number of participating wards	1	4	2	3
Total number of beds in all participating wards	46	64	36	76
Total number of single rooms in all participating wards	0	16	15	16
Total number of physicians on all participating wards during regular working hours	8	12	6	10
Total number of nurses on all participating wards during regular morning shifts	5	8	6	14
Is there a hand disinfectant suspender in each patient room?	Yes	Yes	Yes	Yes

NCP: no contact precautions; SCP: single room contact precautions including gloves, gowns and single room accommodation; VRE: vancomycin-resistant enterococci
* German Commission for Hospital Hygiene and Infection Prevention

Text S1: Description of multimodal hand hygiene program

In order to eliminate the most common denominator in transmitting nosocomial infections, poor hand hygiene- compliance, a hand hygiene program based on the WHO multimodal strategy “Save Lives – Clean Your Hands” was established at all study sites.

Sites received a questionnaire regarding the local infrastructure, which was designed based on the WHO-template. If changes were applied during the study period, the centers were asked to resubmit this document.

Medical staff including physicians, nurses, and medical students were trained in hand hygiene twice during the study. To this end, all sites were provided with slide shows for lectures and seminars, but site-specific training documents were accepted as well. Additionally, physicians and nursing staff were accompanied during their daily working routine for training on the job. On these occasions, feedback was given orally and difficult situations were discussed.

For “reminders in the workspace”, posters, stickers and other visual cues were distributed across all participating wards. Sites were again allowed to use local or provided material, but presence of reminders had to be checked.

During the study, compliance observations were undertaken every 3 months. During each observation, trained staff at each site was asked to observe approximately 150 indications for hand hygiene according to the WHO 5 moments for hand hygiene during ward routine of nurses, physicians and paramedical staff. A standardized monitoring form was used to document number of correctly performed hand hygiene indications and missing ones. These forms were filed for subsequent evaluation.

Finally, to ensure the importance of the program was conveyed, the department director and nursing staff executive were asked to address their staff in a personal letter. The setup of the program with the timeframe is depicted in Table S2.

Table S2: Hand Hygiene Programme Modules

WHO-Module	Content	Timeframe
**System change**	Questionnaire regarding the local infrastructure	Week 1
**Training/Education**	Training seminars of all medical and nursing staff	Week 1-2<br>Month 6
	Training on the job in all new centers, including feedback but no documentation	Month 1
**Evaluation and feedback**	Compliance measurements with monitoring forms including approx. 150 indications across all occupational groups	Month 2-3<br>Month 5-6<br>Month 8-9<br>Month 11-12
**Reminders in the workspace**	"5 hand hygiene indications" poster (translated), sticker, etc.	Continuously
**Institutional safety climate**	Letter from the department director and the nursing staff executive	At initiation

Text S2: Statistical analysis

Patients without any screening performed during their hospitalisation(s) were excluded from the analyses. Furthermore, in patients with only the last hospitalisation without any screening, only this hospitalisation was excluded. Unscreened in between-hospitalisations (between hospitalisations with screening performed) were determined as negative in case of consecutive negativity or lead to censoring of the patient in case of consecutive positivity.

Distribution of data within groups, both at hospitalisation level (counting each hospitalisation separately) and patient level, was described as absolute numbers plus percentage, mean, median and interquartile range (IQR), as appropriate. Comparisons between groups were done using Pearson's chi-square test, Fisher's exact test or Mann-Whitney-U tests.

For primary and secondary endpoints (rates of haVRE, VRE colonisation, VRE BSI and patient-to-patient transmission of VRE), differences in the respective rates comparing NCP and SCP were shown using risk ratios (RR) and 95% confidence intervals (CI). As sensitivity analysis, the primary endpoint of haVRE was assessed in the subgroup of patients and their hospitalisations with at least two screening samples taken.

We used a Fine-Gray distribution hazard regression model to estimate the effect of covariates on the subdistribution hazard ratio (SHR) for haVRE, accounting for the competing risks (last discharge within study period, death, community acquired VRE in the next hospitalisation) (2). We considered single- and multiple-record data (multiple hospitalisations) per subject in our regression model. Subjects and hospitalisations with VRE colonisation at admission or during previous hospitalisations within the study period were excluded. The Fine-Gray distribution hazard regression model was compiled by using STATA (Stata Statistical Software: Release 14. College Station, TX: StataCorp LP), command stcrreg. All variables were selected based on previously identified risk factors for VRE (3-5) and expected demographic differences between groups. Hand hygiene compliance at site level was retrospectively included due to observed differences between sites. Of note, the study was not designed to assess hand hygiene compliance at patient level. Age and underlying disease were assessed from the first hospitalisation during the study period. Categories of age were determined based on even distribution of cases and events. Categories of underlying disease were determined based on clinical meaningfulness and anticipated similar risk for haVRE. Variables and their variations were the following:

- Site group (NCP versus SCP) or site (1-4)
- Age in three categories (≤40; 41-60; >60 years)
- Sex (male; female)
- Underlying haematological disease in four categories (acute leukaemia, lymphoma, solid tumor, other)

For patient level, exposure to antimicrobials and hand hygiene compliance were included as time-varying variables:

- Exposure to antimicrobials with the following variations:
    - Exposure to a certain antimicrobial class or multiple classes during study hospitalisations until current hospitalisation. This was defined as having received any antimicrobial drug during one of the previous or the current hospitalisations. This variables were able to change for each hospitalisation ("was patient x exposed to antimicrobial class y until now?").
    - Exposure to any antimicrobial during study hospitalisations until current hospitalisation
    - Length of exposure to a certain antimicrobial class or multiple classes during study hospitalisations until current hospitalisation in days ("How many days did patient x receive antimicrobial class y until now?")
    - Length of exposure to any antimicrobial during study hospitalisations until current hospitalisation in days
    - Length of exposure to a certain antimicrobial class or multiple classes during study hospitalisations until current hospitalisation in three categories (no exposure; ≤7 cumulative days until now; longer exposure). These categories were based on the documented exposure for each class and each hospitalisation in days.

  - Length of exposure to any antimicrobial during study hospitalisations until current hospitalisation in three categories (see above).
- Compliance with hand hygiene at respective site during the current hospitalisation in the categories ≤75% and >75%. Compliance rates were taken from the results of the last available compliance measurements performed as part of the hand hygiene program in the study.

As sensitivity analysis, the regression model was repeated for hospitalisation level with the same variables except for exposure to antimicrobials and compliance with hand hygiene being included not as time-varying covariates:

- Exposure to antimicrobials with the following variations:
  - Exposure to a certain antimicrobial class or multiple classes in this hospitalisation
  - Exposure to any antimicrobial class in this hospitalisation
  - Length of exposure to a certain antimicrobial class or multiple classes in this hospitalisation in days
  - Length of exposure to any antimicrobial in this hospitalisation in days
  - Length of exposure to a certain antimicrobial class or multiple classes in this hospitalisation in three categories (no exposure; ≤7 days; >7 days)
  - Length of exposure to any antimicrobial in this hospitalisation in three categories (see above)
- Compliance with hand hygiene at respective site during this hospitalisation in the categories ≤75% and >75%.

Within our explorative approach, we considered all above described potential clinical and sociodemographic confounders to adjust our final multivariable model using the Enter method, independent of the p-value in the univariate analysis. We selected our final model by considering the model characteristics (Akaike information criterion and Bayesian information criterion) and clinical meaningfulness (rather differentiation of antimicrobial classes than summarized as “any”). The final model was adjusted for site group, age in three categories, sex, underlying disease in four categories, exposure to different antimicrobial classes assessed cumulatively until current hospitalisation, and hand hygiene compliance. Alternative models are included as sensitivity analyses.

As an exploratory analysis, the regression was repeated with the endpoint of patient-to-patient transmission instead of haVRE with identical variables and their variations. Competing risks were last discharge within study period, death, community acquired VRE in the next hospitalisation, haVRE but not transmission receiver according to analysis.

Table S3: Hospitalisation characteristics and length of stay until VRE acquisition*

	NCP<br>*n*=2435	SCP<br>*n*=3023	p value
Length of stay in days: Median (IQR)	8 (5-17)	7 (4-14)	<0.001$
Exposure to any antimicrobial class (%)	1422 (58.4)	1295 (42.8)	<0.001§
Exposure to antimicrobials active against VRE (lipopeptides and oxazolidones) (%)			<0.001†
None	2388 (98.1)	2882 (95.3)	
≤ 7 days	36 (1.5)	59 (2.0)	
> 7 days	11 (0.5)	82 (2.7)	
Exposure to cephalosporins (%)			<0.001†
None	2258 (92.7)	2872 (95.0)	
≤ 7 days	149 (6.1)	107 (3.5)	
> 7 days	28 (1.1)	44 (1.5)	
Exposure to fluoroquinolones (%)			<0.001†
None	2240 (92.0)	2462 (81.4)	
≤ 7 days	151 (6.2)	350 (11.6)	
> 7 days	44 (1.8)	211 (7.0)	
Exposure to glycopeptides (%)			0.621†
None	2293 (94.2)	2856 (94.5)	
≤ 7 days	104 (4.3)	115 (3.8)	
> 7 days	38 (1.6)	52 (1.7)	
Exposure to other antimicrobial class (%)			<0.001†
None	1078 (44.3)	1861 (61.6)	
≤ 7 days	872 (35.8)	738 (24.4)	
> 7 days	485 (19.9)	424 (14.0)	
Length of stay until VRE acquisition*: Median (IQR)	14 (8-20)	12 (8-17.5)	0.123$

VRE: vancomycin-resistant enterococci; NCP: no contact precautions; SCP: single room contact precautions; IQR: Interquartile range;

$ Mann-Whitney U Test

§ Fisher's exact test

† Pearson's χ^2 test

* Only hospitalisations with hospital-acquired colonisation or infection with VRE included in calculation.

Table S4: Characteristics of 17 cases with VRE bloodstream infections including attributable mortality

Site	ST	BSI during hospitalisation on study ward?	Death during study period or within 60 days of BSI*	Time BSI to death in days	Death attributable to VRE BSI**	Note
NCP1	17	x	yes	105	no	No further VRE BSI shortly before death.
NCP2	117	yes	no			
	117	yes	no			
	117	no	no			
	117	no	no			
	203	no	no			
	117	no	no			
	117	no	yes	11	no	Died due to sepsis in neutropenia with ongoing BSI due to *E. coli*. Detection of VRE only in two blood cultures 11 days prior death.
	80	yes	no			
	203	no	yes	53	no	No further VRE BSI shortly before death.
SCP1	192	yes	no			
	203	no	no			
	117	no	no			
SCP2	117	yes	no			
	117	yes	no			
	132	yes	yes	48	no	No further VRE BSI shortly before death.
	80	no	yes	6	yes	Died due to sepsis in neutropenia and progressive underlying disease, no alternative pathogen detected.

VRE: vancomycin-resistant enterococci; NCP: no contact precautions; SCP: single room contact precautions; BSI: bloodstream infection; ST: sequence type

*For BSI cases occurring during the last two month of the observational period of the study, hospital records were checked retrospectively to determine mortality within 60 days of onset of BSI.

** Attributable mortality in patients with VRE BSI was defined as death within seven days of onset of VRE BSI or of last positive VRE blood culture in case of ongoing BSI.

Table S5: Distribution of *van* genes determined by WGS (n=505)

***Van* gene**	**NCP-sites; n (%)**	**SCP sites; n (%)**	**Total; n (%)**
vanA	83 (28.9)	28 (12.8)	111 (22.0)
vanB	203 (70.7)	190 (87.1)	393 (77.8)
vanA+B	1 (0.3)	0	1 (0.2)
Total	287	218	505

WGS: whole genome sequencing; NCP: no contact precautions; SCP: single room contact precautions

Table S6: Distribution of multi-locus sequence types derived from WGS (n=505)

**Sequence type**	**NCP-sites; n (%)**	**SCP sites; n (%)**	**Total; n (%)**
ST-17	52 (18.1)	17 (7.8)	69 (13.7)
ST-18	0	7 (3.2)	7 (1.4)
ST-78	0	1 (0.5)	1 (0.2)
ST-80	26 (9.1)	65 (29.8)	91 (18.0)
ST-117	155 (54.0)	101 (46.3)	256 (50.7)
ST-132	0	3 (1.4)	3 (0.6)
ST-192	15 (5.2)	7 (3.2)	22 (4.4)
ST-203	37 (12.9)	5 (2.3)	42 (8.3)
ST-262	1 (0.3)	11 (5.0)	12 (2.4)
ST-323	1 (0.3)	1 (0.5)	2 (0.4)
Total	287	218	505

WGS: whole genome sequencing; NCP: no contact precautions; SCP: single room contact precautions

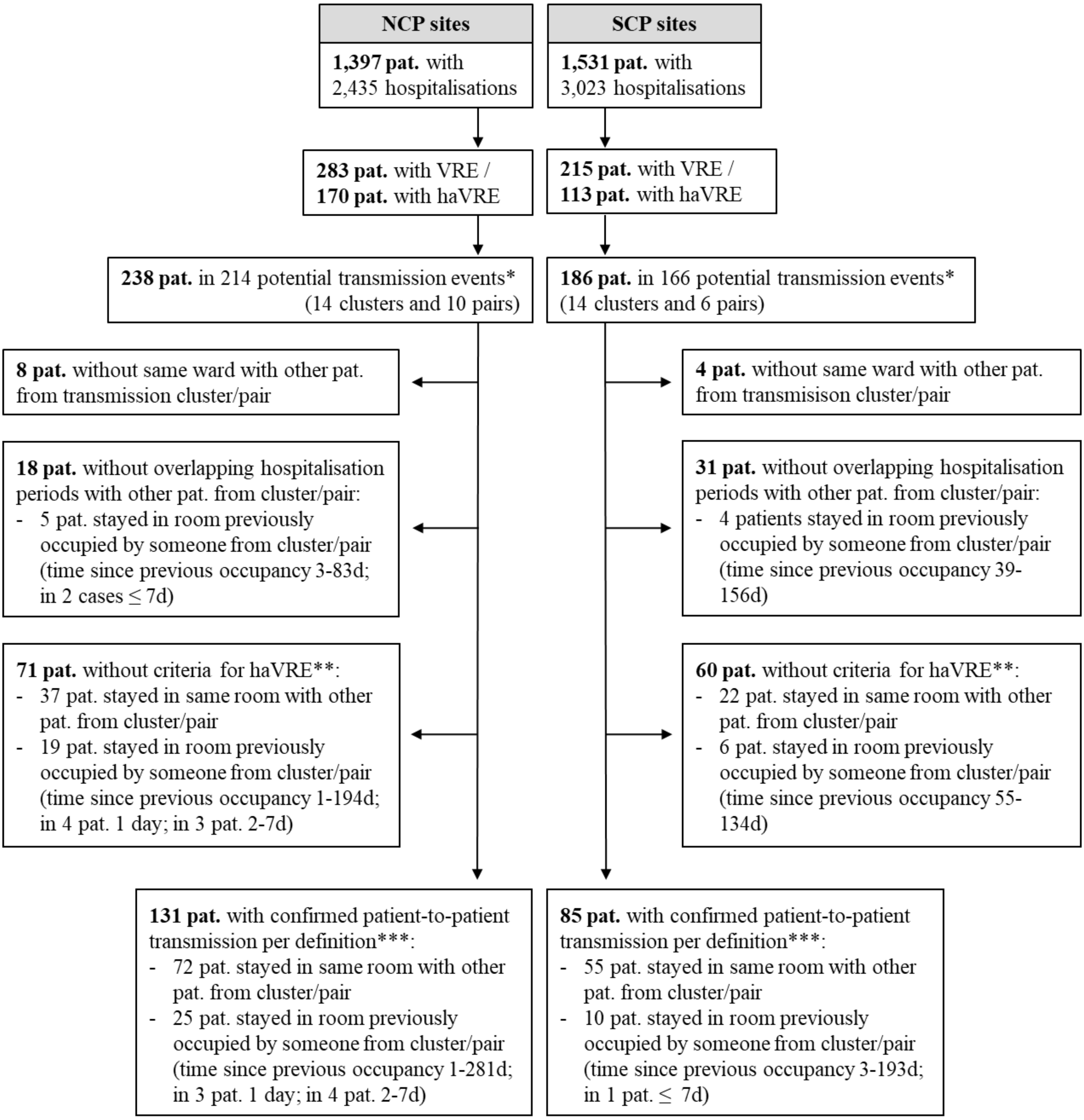

Figure S1: Flow chart of stepwise assessment of patient-to-patient transmissions of VRE
* For each potential transmission event, at least two patients need to be involved. Example: a pair of two patients with isolates showing close relatedness determined by cgMLST, one transmission event could have occurred. In a cluster of three patients, two separate events could have occurred. At NCP sites, 10 pairs and 14 clusters involving 238 patients were observed, which would allow a maximum of 214 separate transmission events. At SCP sites, we observed 6 pairs and 14 clusters involving a total of 186 patients, which would allow a maximum of 166 separate transmission events.
**haVRE: in-hospital acquisition of VRE defined as a screening or clinical culture obtained >72h after admission yielding VRE in a patient with a negative admission screening.
*** Patient-to-patient transmission was defined as the isolation of closely related isolates from ≥2 patients in the same ward with overlapping hospitalisation periods and with at least one patient with haVRE for each transmission event.
VRE: vancomycin-resistant enterococci*;* NCP: no contact precautions; SCP: single room contact precautions; pat.: patients; WGS: whole genome sequencing; cgMLST: core genome multi-locus sequence typing;

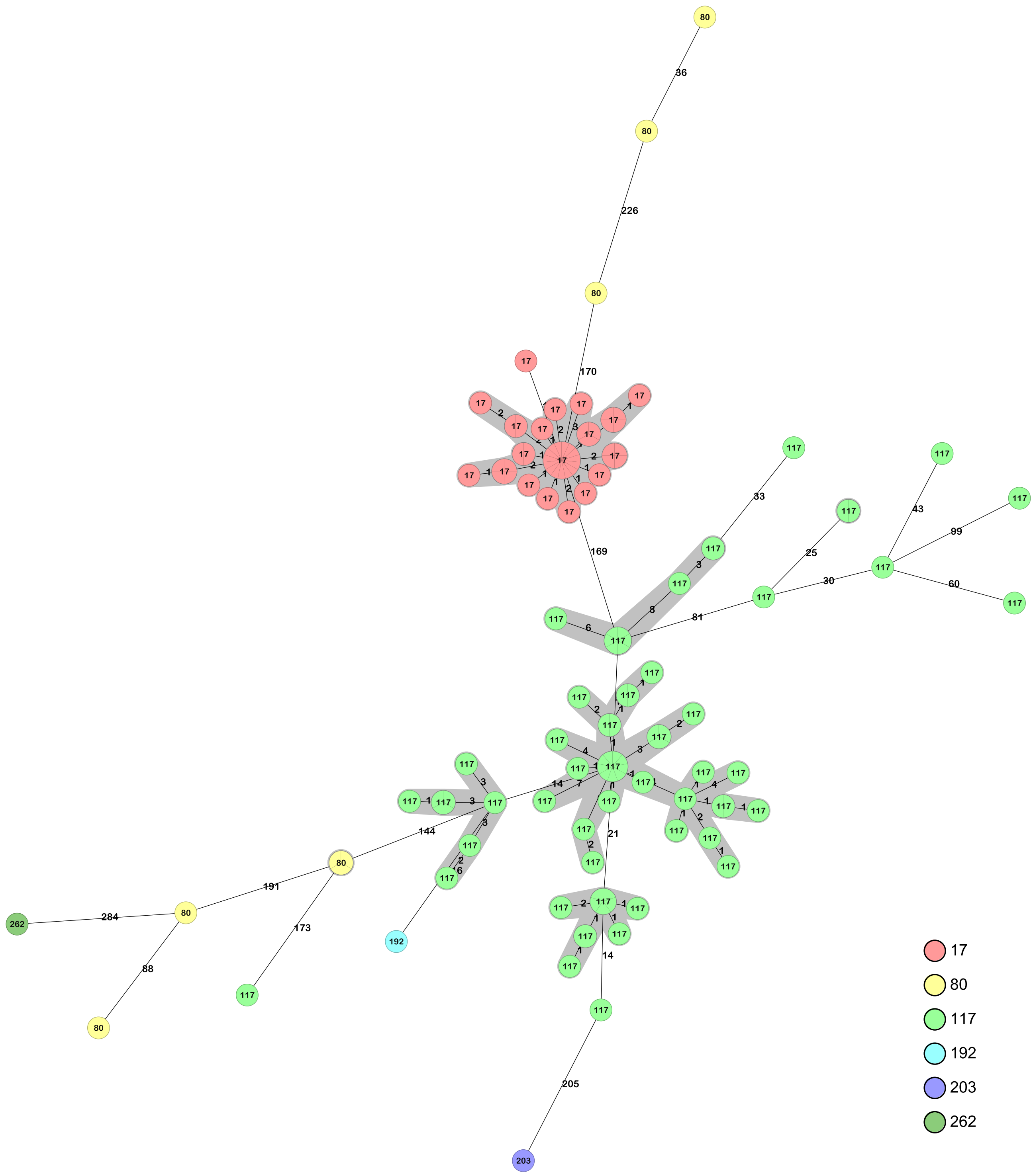

Figure S2a: Minimum spanning tree of all surveillance isolates subjected to WGS and cgMLST showing their relatedness at site NCP1

Undivided circles represent one isolate from an individual patient, circles with division lines represent more than one isolate from different patients with 0 allele difference. Labelling and colour of the circles show sequence type determined by 7-loci sequence typing. Numbers between circles indicate allele differences. A grey connection line or halo shows close relatedness by cgMLST. WGS: whole genome sequencing; cgMLST: core genome multi-locus sequence typing; NCP: no contact precautions

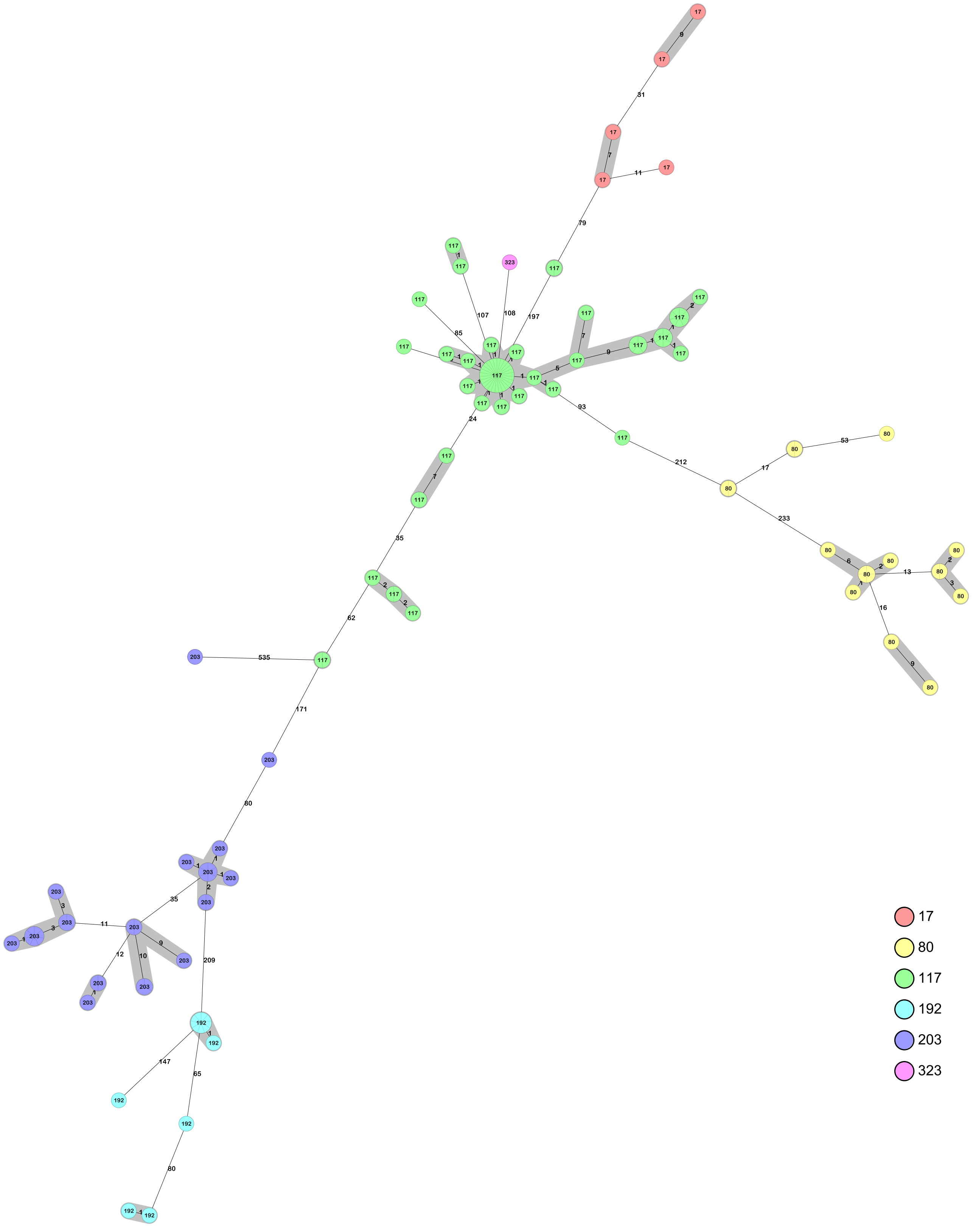

Figure S2b: Minimum spanning tree of all surveillance isolates subjected to WGS and cgMLST showing their relatedness at site NCP2

Undivided circles represent one isolate from an individual patient, circles with division lines represent more than one isolate from different patients with 0 allele difference. Labelling and colour of the circles show sequence type determined by 7-loci sequence typing. Numbers between circles indicate allele differences. A grey connection line or halo shows close relatedness by cgMLST. WGS: whole genome sequencing; cgMLST: core genome multi-locus sequence typing; NCP: no contact precautions

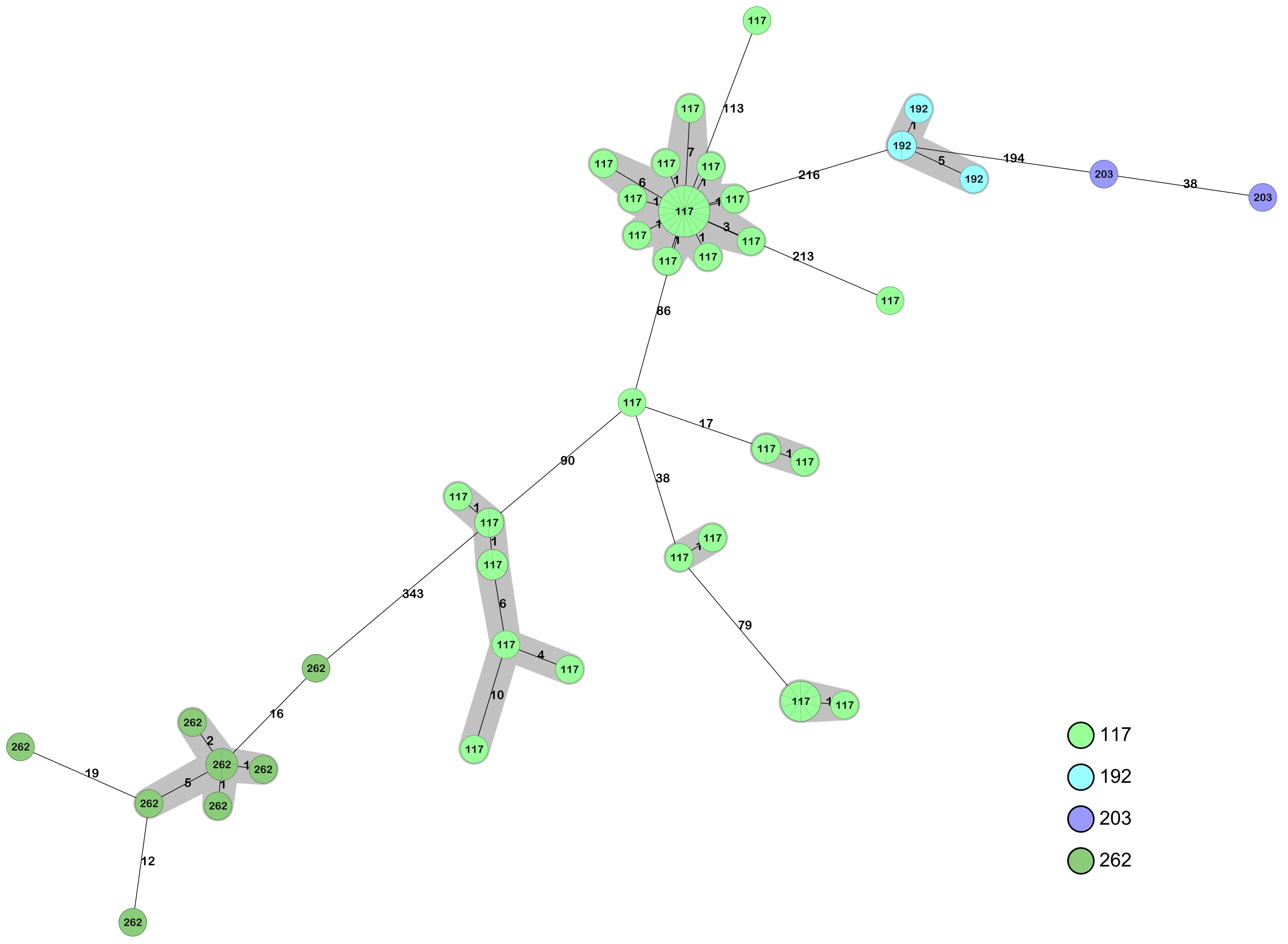

Figure S2c: Minimum spanning tree of all surveillance isolates subjected to WGS and cgMLST showing their relatedness at site SCP1

Undivided circles represent one isolate from an individual patient, circles with division lines represent more than one isolate from different patients with 0 allele difference. Labelling and colour of the circles show sequence type determined by 7-loci sequence typing. Numbers between circles indicate allele differences. A grey connection line or halo shows close relatedness by cgMLST. WGS: whole genome sequencing; cgMLST: core genome multi-locus sequence typing; SCP: single room contact precautions.

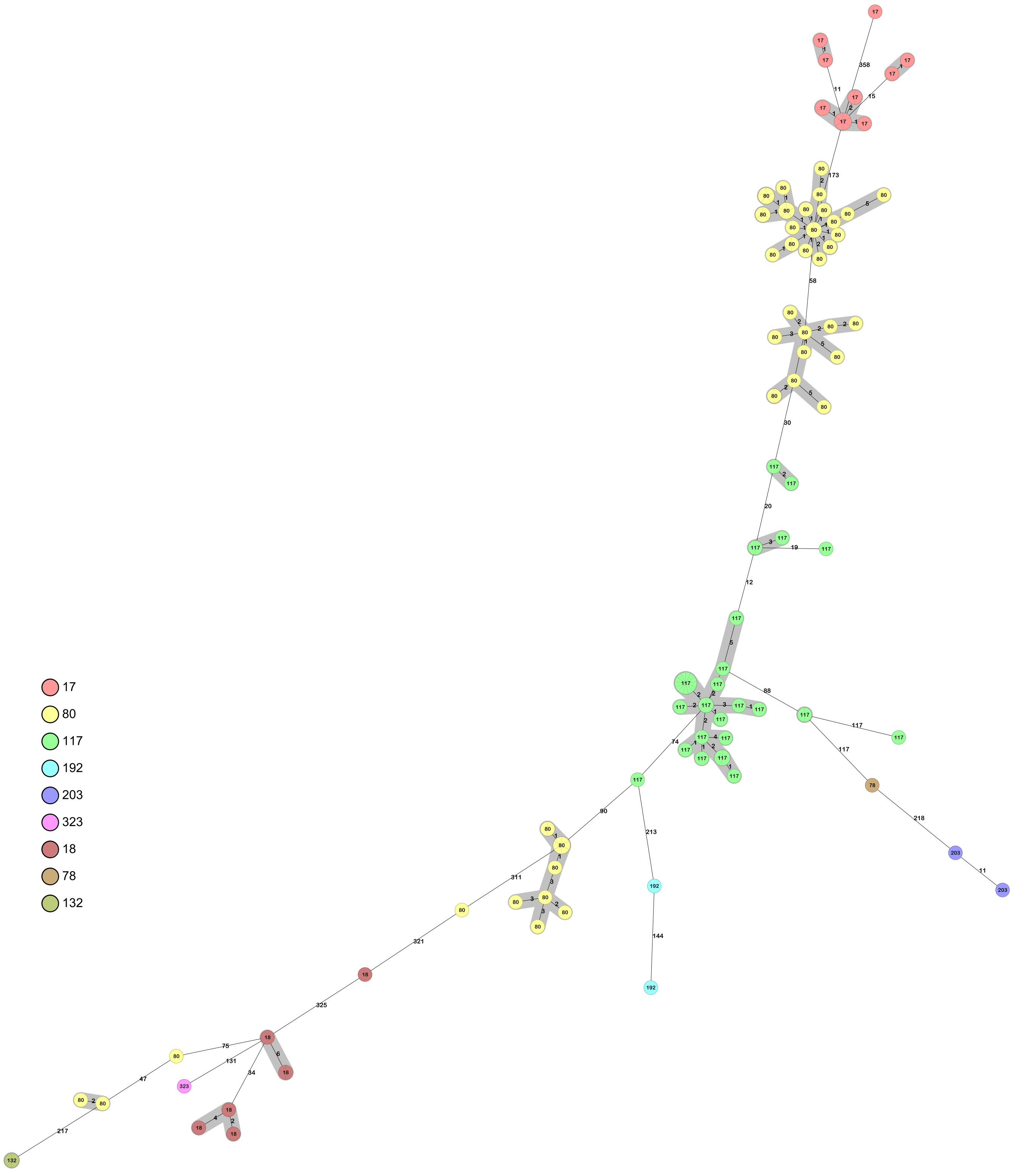

Figure S2d: Minimum spanning tree of all surveillance isolates subjected to WGS and cgMLST showing their relatedness at site SCP2

Undivided circles represent one isolate from an individual patient, circles with division lines represent more than one isolate from different patients with 0 allele difference. Labelling and colour of the circles show sequence type determined by 7-loci sequence typing. Numbers between circles indicate allele differences. A grey connection line or halo shows close relatedness by cgMLST. WGS: whole genome sequencing; cgMLST: core genome multi-locus sequence typing; SCP: single room contact precautions.

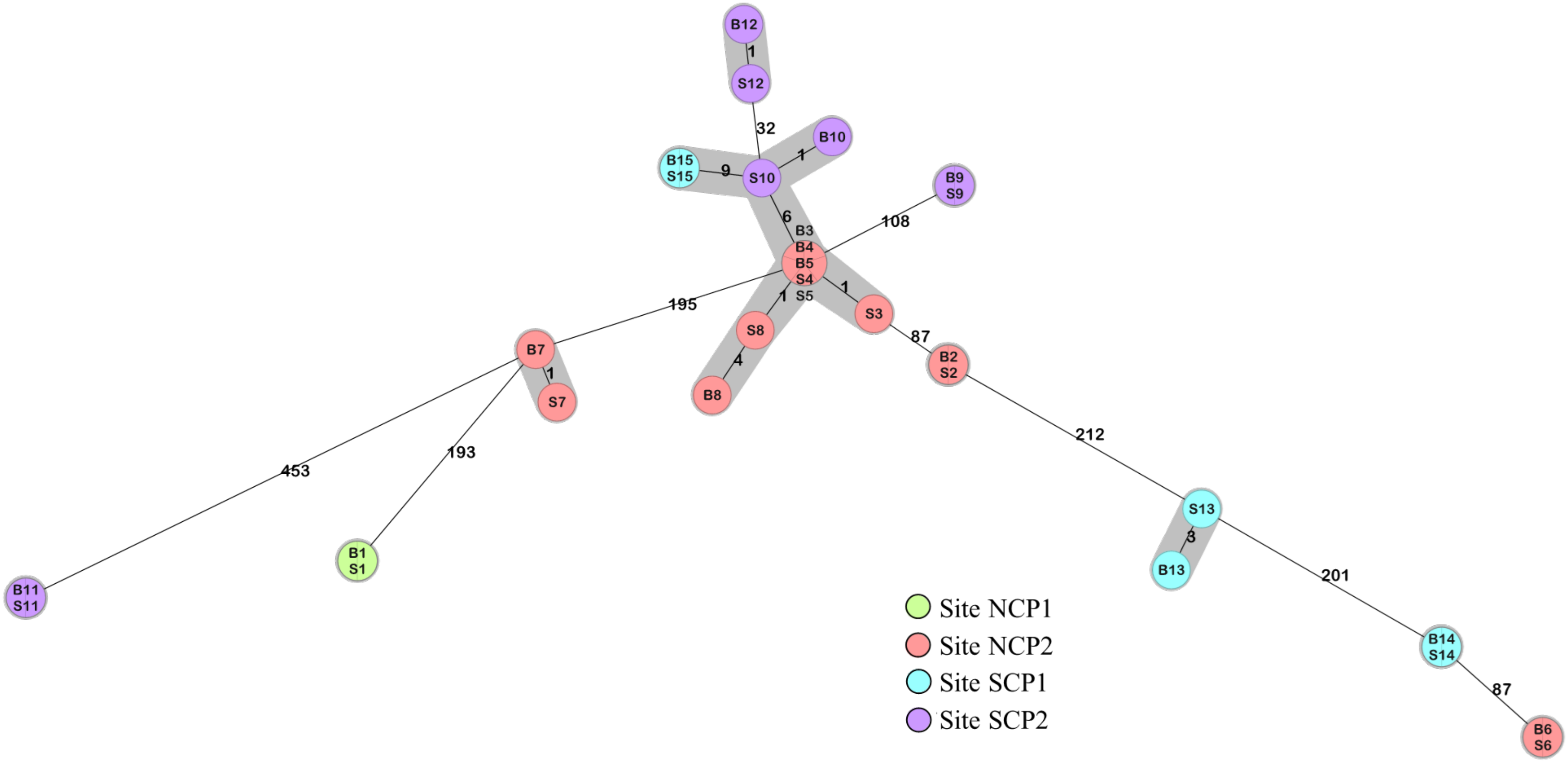

Figure S3: Relatedness of bloodstream and corresponding surveillance isolates from five patients as determined by cgMLST
Each circle represents one isolate from an individual patient with VRE bloodstream infection. Circles with a vertical division line indicate two samples with 0 allele differences. Numbers between circles represent allele differences. Labelling indicates bloodstream (B) and surveillance (S) isolates from each patient (numbers 1-5). Colour of circles indicates site.
cgMLST: core genome multi-locus sequence typing; VRE: vancomycin-resistant enterococci*;* NCP: no contact precautions; SCP: single room contact precautions

Table S7: Details of identified clusters and pairs with close-relatedness among VRE isolates of each site

Site		Cluster	No. of involved isolates	No. of confirmed transmissions*	No. of transmissons sharing room*	No. of involved wards	Time period	ST	Multi-site cluster**	No. of inter-site relatedness**
NCP	1	1A	45	21	16	2	Jan-Dec	17	i	3
		1B	10	6	0	2	Mar-Nov	117	e	4
		1C	33	20	13	2	Jan-Dec	117	e	2
		1D	8	5	4	2	Aug-Dec	117	d	6
		1E	3	3	2	1	Sep-Oct	80	c	1
		1 pair	2	0	0	1	Oct-Nov	117		
	2	2A	59	43	23	2	Mar-Dec	117	d	3
		2B	7	2	0	2	Oct-Dec	80		
		2C	4	1	1	2	Jul-Oct	80		
		2D	3	0	0	2	Apr-May	117	e	2
		2E	4	2	2	2	Feb-Apr	117		
		2F	11	8	1	2	Oct-Dec	203		
		2G	18	8	3	2	Jan-Sep	203	a	5
		2H	3	2	2	2	Aug-Sep	203		
		2I	10	8	4	2	Feb-Jun	192		
		9 pairs	18	2	1	1 each	#	#	f,b,g	1 each
	Total	14 clusters 10 pairs	238	131	72	-	Jan-Dec	-	-	-
SCP	3	3A	9	1	0	2	Jan-Aug	117	h	2
		3B	11	6	3	2	Mar-Sep	117		
		3C	3	1	0	1	Feb-Apr	117		
		3D	28	19	16	2	Oct-Dec	117	d	1
		3E	4	1	1	1	Mar-Nov	192		
		3F	8	7	6	1	Jun-Sep	262		
		1 pair	2	1	0	1	Feb-Mar	117		
	4	4A	14	6	1	2	Feb-Jul	17	i	1
		4B	35	20	13	2	Feb-Dec	80		
		4C	11	0	0	3	Jan-Dec	80		
		4D	3	2	2	1	Mar-Oct	117	e	1
		4E	29	12	10	3	Apr-Dec	117	e	9
		4F	13	5	2	2	Feb-Nov	80	c	1
		4G	3	1	0	2	Mar-Apr	18		
		4H	3	1	0	2	Jul	18		
		5 pairs	10	2	1	1 each	§	§	d, g, i	1 each
	Total	14 clusters /6 pairs	186	85	55	-	Jan-Dec	-	-	-

VRE: Vancomycin-resistant enterococci; NCP: no contact precautions; SCP: single room contact precautions; ST: 7-loci sequence type;

*Patient-to-patient transmission was defined as overlapping hospitalisation period on same ward, at least one patient per transmission event with haVRE and close relatedness demonstrated by molecular typing (maximum of 10 allele differences by cgMLST). Hospitalisation in the same room at the same time was not a prerequisite, but was assessed.

**Multi-site clusters contain closely related isolates as determined by cgMLST (maximum of 10 allele differences) from at least two different sites. See figure 2 for illustration. Isolates with inter-site relatedness were counted.

#Isolates were detected during the following time periods: Jan-Mar (ST-117)/ 2xFeb-Mar (ST-80; ST-192)/ Mar (ST-17)/ Mar-Apr (ST-117)/ Apr (ST-117)/ May-Sep (ST-17)/ Jun-Jul (ST-80)/ Jun-Aug (ST-80)

§ Isolates were detected during the following time periods: Jan-Feb (ST-17)/ Feb (ST-117)/ Apr-Sep (ST-117)/ Aug-Sep (ST-80)/ Dec (ST-132)

Table S8: Details of multi-site clusters with close-relatedness among VRE from different sites

Cluster	Sites involved	Total no. of isolates	No. of clusters/isolate pairs involved	No. of isolates not part of a cluster/pair	Time period	No. of inter-site relatedness*	ST
a	1,2,4	21	1 cluster	3	Jan-Dec	5	203
b	1,2	3	1 pair	1	Mar-Sep	1	117
c	1,4	16	2 clusters	0	Feb-Dec	1	80
d**	1,2,3,4	100	3 clusters, 2 pairs	0	Mar-Dec	8	117
e**	1,2,4	78	5 clusters	0	Feb-Dec	12	117
f	2,3	3	1 pair	1	Jan-Oct	1	117
g	2,4	4	1 pair	2	Feb-Apr	1	117
h	1,3,4	11	1 cluster	2	Jan-Aug	2	117
i	1,4	61	2 clusters	2	Jan-Dec	3	17
**Total*** **	1-4	297/494	14/28 clusters 6/16 pairs	11	Jan-Dec	34	-

VRE: Vancomycin-resistant enterococci; NCP: no contact precautions; SCP: single room contact precautions; ST: 7-loci sequence type;
*Multi-site clusters contain closely related isolates as determined by cgMLST (maximum of 10 allele differences) from at least two different sites. See figure 2 for illustration. Isolates with inter-site relatedness were counted.
**An isolate of each cluster d and e are only 12 alleles apart.
***Total numbers of isolates, clusters and pairs involved in multi-site complexes in reference to total number of characterised isolates and observed clusters and pairs within sites.

Tables S9a-i: Competing risk analysis for hospital-acquired VRE colonisation or bloodstream infection at the patient level: univariate and multivariate regression – Sensitivity analyses (variations highlighted in yellow)

Table S9a: Variation with separate sites (patient level)

	**Univariate analysis**			**Multivariate analysis:**		
**Variable**	**SHR***	**95% CI**	**p value**	**SHR***	**95% CI**	**p value**
Site						
SCP2						
SCP1	1.48	1.02-2.15	0.041	2.00	1.08-3.71	0.027
NCP1	2.45	1.76-3.41	<0.001	2.76	1.91-3.99	<0.001
NCP2	1.65	1.21-2.25	0.001	1.26	0.62-2.55	0.522
Age						
≤40 years						
41-60 years	1.28	0.87-1.88	0.215	1.22	0.82-1.81	0.323
>60 years	1.27	0.88-1.82	0.199	1.29	0.89-1.87	0.182
Sex						
Female						
Male	1.22	0.96-1.55	0.105	1.20	0.94-1.54	0.135
Underlying haematological disease categorised						
Other						
Acute leukaemia	3.17	2.04-4.92	<0.001	1.96	1.22-3.15	0.005
Lymphoma	1.84	1.19-2.85	0.006	1.26	0.81-1.97	0.311
Solid tumor	1.19	0.73-1.93	0.486	1.14	0.70-1.86	0.605
Exposure to antimicrobials active against VRE[#]						
No						
Yes	0.95	0.61-1.49	0.838	0.80	0.49-1.31	0.378
Exposure to cephalosporins[#]						
No						
Yes	2.34	1.79-3.07	<0.001	1.74	1.24-2.45	0.001
Exposure to fluoroquinolones[#]						
No						
Yes	1.53	1.20-1.95	0.001	1.48	1.10-1.99	0.009
Exposure to glycopeptides[#]						
No						
Yes	2.12	1.61-2.79	<0.001	1.44	1.05-1.98	0.025
Exposure to other antimicrobials[#]						
No						
Yes	4.95	3.37-7.27	<0.001	5.13	3.16-8.32	<0.001
Compliance with hand hygiene at respective site during this hospitalisation[#]						
>75%						
Up to 75%	1.35	1.07-1.71	0.010	1.47	0.80-2.70	0.211

VRE: vancomycin-resistant enterococci; NCP: no contact precautions; SCP: single room contact precautions; SHR: subdistribution hazard ratio; CI: confidence interval

[#]Exposure to antimicrobials and compliance with hand hygiene were included as time-varying covariate and could change for each hospitalisation. Exposure to antimicrobials was assessed cumulatively until current hospitalisation.

*SHR and 95% CI for SHR obtained from the Fine-Gray model.

Table S9b: Variation with exposure to any antimicrobial in days (patient level)

	Univariate analysis			Multivariate analysis:		
**Variable**	**SHR***	**95% CI**	**p value**	**SHR***	**95% CI**	**p value**
Site group						
SCP						
NCP	1.71	1.35-2.17	<0.001	1.66	1.20-2.31	0.003
Age						
≤40 years						
41-60 years	1.28	0.87-1.88	0.215	1.27	0.86-1.88	0.232
>60 years	1.27	0.88-1.82	0.199	1.29	0.89-1.87	0.179
Sex						
Female						
Male	1.22	0.96-1.55	0.112	1.23	0.97-1.58	0.092
Underlying disease categorised						
Other						
Acute leukaemia	4.80	3.09-7.46	<0.001	3.68	2.31-5.85	<0.001
Lymphoma	2.39	1.55-3.70	<0.001	1.89	1.21-2.94	0.005
Solid tumor	1.25	0.77-2.04	0.368	1.13	0.69-1.84	0.623
Length of exposure to any antimicrobial in days[#]**	1.01	1.01-1.02	<0.001	1.01	1.004-1.013	<0.001
Compliance with hand hygiene at respective site during this hospitalisation[#]						
>75%						
Up to 75%	1.42	1.13-1.80	0.003	1.12	0.82-1.54	0.467

VRE: vancomycin-resistant enterococci; NCP: no contact precautions; SCP: single room contact precautions; SHR: subdistribution hazard ratio; CI: confidence interval

[#]Exposure to antimicrobials and compliance with hand hygiene were included as time-varying covariate and could change for each hospitalisation. Exposure to antimicrobials was assessed cumulatively until current hospitalisation.

*SHR and 95% CI for SHR obtained from the Fine-Gray model

**Days of exposure to different antibiotic classes were summed up irrespective if given in parallel or sequentially.

Table S9c: Variation with exposure to any antimicrobial irrespective of length (patient level)

	Univariate analysis			Multivariate analysis:		
**Variable**	**SHR***	**95% CI**	**p value**	**SHR***	**95% CI**	**p value**
Site group						
SCP						
NCP	1.71	1.35-2.17	<0.001	1.26	0.90-1.76	0.171
Age						
≤40 years						
41-60 years	1.28	0.87-1.88	0.215	1.25	0.84-1.86	0.264
>60 years	1.27	0.88-1.82	0.199	1.28	0.88-1.85	0.199
Sex						
Female						
Male	1.22	0.96-1.55	0.112	1.26	0.99-1.61	0.063
Underlying disease categorised						
Other						
Acute leukaemia	4.80	3.09-7.46	<0.001	3.13	2.00-4.91	<0.001
Lymphoma	2.39	1.55-3.70	<0.001	1.49	0.96-2.34	0.078
Solid tumor	1.25	0.77-2.04	0.368	1.33	0.81-2.17	0.259
Exposure to any antimicrobial[#]						
No						
Yes	9.08	5.70-14.47	<0.001	7.46	4.59-12.12	<0.001
Compliance with hand hygiene at respective site during this hospitalisation[#]						
>75%						
≤75%	1.42	1.13-1.80	0.003	1.27	0.92-1.74	0.144

VRE: vancomycin-resistant enterococci*;* NCP: no contact precautions; SCP: single room contact precautions; SHR: subdistribution hazard ratio; CI: confidence interval

[#]Exposure to antimicrobials and compliance with hand hygiene were included as time-varying covariate and could change for each hospitalisation. Exposure to antimicrobials was assessed cumulatively until current hospitalisation.

*SHR and 95% CI for SHR obtained from the Fine-Gray model

Table S9d: Variation with length of exposure to any antimicrobials in categories (patient level)

	Univariate analysis			Multivariate analysis:		
**Variable**	**SHR***	**95% CI**	**p value**	**SHR***	**95% CI**	**p value**
Site group						
SCP						
NCP	1.71	1.35-2.17	<0.001	1.33	0.95-1.85	0.096
Age						
≤40 years						
41-60 years	1.28	0.87-1.88	0.215	1.23	0.83-1.83	0.304
>60 years	1.27	0.88-1.82	0.199	1.29	0.89-1.87	0.186
Sex						
Female						
Male	1.22	0.96-1.55	0.112	1.25	0.98-1.59	0.071
Underlying disease categorised						
Other						
Acute leukaemia	4.80	3.09-7.46	<0.001	2.60	1.64-4.14	<0.001
Lymphoma	2.39	1.55-3.70	<0.001	1.37	0.87-2.14	0.174
Solid tumor	1.25	0.77-2.04	0.368	1.24	0.76-2.03	0.383
Length of exposure to any antimicrobial in categories (different antibiotic classes cumulated) #**						
None						
≤7 days until now	5.63	3.39-9.35	<0.001	5.16	3.08-8.64	<0.001
>7 days until now	11.79	7.36-18.88	<0.001	9.30	5.64-15.32	<0.001
Compliance with hand hygiene at respective site during this hospitalisation#						
>75%						
≤75%	1.42	1.13-1.80	0.003	1.24	0.90-1.70	0.189

VRE: vancomycin-resistant enterococci; NCP: no contact precautions; SCP: single room contact precautions; SHR: subdistribution hazard ratio; CI: confidence interval

#Exposure to antimicrobials and compliance with hand hygiene were included as time-varying covariate and could change for each hospitalisation. Exposure to antimicrobials was assessed cumulatively until current hospitalisation.

*SHR and 95% CI for SHR obtained from the Fine-Gray model

**Days of exposure to different antibiotic classes were summed up irrespective if given in parallel or sequentially.

Table S9e: Variation with length of exposure to antimicrobials active or not active against VRE in days (patient level)

	Univariate analysis			Multivariate analysis:		
**Variable**	**SHR***	**95% CI**	**p value**	**SHR***	**95% CI**	**p value**
Site group						
SCP						
NCP	1.71	1.35-2.17	<0.001	1.61	1.16-2.23	0.004
Age						
≤40 years						
41-60 years	1.28	0.87-1.88	0.215	1.28	0.86-1.90	0.231
>60 years	1.27	0.88-1.82	0.199	1.36	0.93-1.98	0.109
Sex						
Female						
Male	1.22	0.96-1.55	0.112	1.23	0.96-1.57	0.099
Underlying disease categorised						
Other						
Acute leukaemia	4.80	3.09-7.46	<0.001	3.54	2.25-5.58	<0.001
Lymphoma	2.39	1.55-3.70	<0.001	1.92	1.23-2.98	0.004
Solid tumor	1.25	0.77-2.04	0.368	1.15	0.71-1.88	0.566
Length of exposure to antimicrobials active against VRE in days (lipopeptides and oxazolidinones) #**	1.01	0.99-1.04	0.286	0.94	0.90-0.98	0.009
Length of exposure to antimicrobials not active against VRE in days#**	1.01	1.01-1.02	<0.001	1.02	1.01-1.02	<0.001
Compliance with hand hygiene at respective site during this hospitalisation#						
>75%						
≤75%	1.42	1.13-1.80	0.003	1.05	0.77-1.43	0.743

VRE: vancomycin-resistant enterococci; NCP: no contact precautions; SCP: single room contact precautions; SHR: subdistribution hazard ratio; CI: confidence interval

#Exposure to antimicrobials and compliance with hand hygiene were included as time-varying covariate and could change for each hospitalisation. Exposure to antimicrobials was assessed cumulatively until current hospitalisation.

*SHR and 95% CI for SHR obtained from the Fine-Gray model

**Days of exposure to different antibiotic classes were summed up irrespective if given in parallel or sequentially.

Table S9f: Variation with exposure to antimicrobials active or not active against VRE irrespective of length (patient level)

	Univariate analysis			Multivariate analysis:		
**Variable**	**SHR***	**95% CI**	**p value**	**SHR***	**95% CI**	**p value**
Site group						
SCP						
NCP	1.71	1.35-2.17	<0.001	1.25	0.89-1.74	0.196
Age						
≤40 years						
41-60 years	1.28	0.87-1.88	0.215	1.26	0.85-1.87	0.256
>60 years	1.27	0.88-1.82	0.199	1.28	0.88-1.86	0.197
Sex						
Female						
Male	1.22	0.96-1.55	0.112	1.26	0.99-1.61	0.062
Underlying disease categorised						
Other						
Acute leukaemia	4.80	3.09-7.46	<0.001	3.21	2.04-5.06	<0.001
Lymphoma	2.39	1.55-3.70	<0.001	1.51	0.96-2.37	0.072
Solid tumor	1.25	0.77-2.04	0.368	1.33	0.81-2.18	0.254
Exposure to antimicrobials active against VRE (lipopetides and oxazolidinones) #						
No						
Yes	1.53	0.98-2.37	0.059	0.85	0.52-1.37	0.496
Exposure to antimicrobials not active against VRE#						
No						
Yes	9.08	5.70-14.47	<0.001	7.54	4.64-12.25	<0.001
Compliance with hand hygiene at respective site during this hospitalisation#						
>75%						
≤75%	1.42	1.13-1.80	0.003	1.25	0.91-1.72	0.173

VRE: vancomycin-resistant enterococci; NCP: no contact precautions; SCP: single room contact precautions; SHR: subdistribution hazard ratio; CI: confidence interval

#Exposure to antimicrobials and compliance with hand hygiene were included as time-varying covariate and could change for each hospitalisation. Exposure to antimicrobials was assessed cumulatively until current hospitalisation.

*SHR and 95% CI for SHR obtained from the Fine-Gray model

Table S9g: Variation with categorised length of exposure to antimicrobials active or not active against VRE (patient level)

	Univariate analysis			Multivariate analysis:		
**Variable**	**SHR***	**95% CI**	**p value**	**SHR***	**95% CI**	**p value**
Site group						
SCP						
NCP	1.71	1.35-2.17	<0.001	1.30	0.93-1.81	0.126
Age						
≤40 years						
41-60 years	1.28	0.87-1.88	0.215	1.24	0.83-1.85	0.287
>60 years	1.27	0.88-1.82	0.199	1.29	0.89-1.87	0.182
Sex						
Female						
Male	1.22	0.96-1.55	0.112	1.25	0.98-1.60	0.069
Underlying disease categorised						
Other						
Acute leukaemia	4.80	3.09-7.46	<0.001	2.72	1.71-4.35	<0.001
Lymphoma	2.39	1.55-3.70	<0.001	1.39	0.89-2.19	0.148
Solid tumor	1.25	0.77-2.04	0.368	1.25	0.77-2.04	0.372
Length of exposure to antimicrobials active against VRE in categories (lipopeptides and oxazolidinones) #**						
None						
≤7 days until now	1.47	0.75-2.88	0.260	0.76	0.38-1.53	0.442
>7 days until now	1.57	0.89-2.78	0.118	0.69	0.37-1.27	0.230
Length of exposure to antimicrobials not active against VRE in categories#**						
None						
≤7 days until now	5.71	3.44-9.47	<0.001	5.24	3.13-8.76	<0.001
>7 days until now	11.79	7.36-18.88	<0.001	9.57	5.81-15.76	<0.001
Compliance with hand hygiene at respective site during this hospitalisation#						
>75%						
≤75%	1.42	1.13-1.80	0.003	1.19	0.87-1.63	0.281

VRE: vancomycin-resistant enterococci; NCP: no contact precautions; SCP: single room contact precautions; SHR: subdistribution hazard ratio; CI: confidence interval

#Exposure to antimicrobials and compliance with hand hygiene were included as time-varying covariate and could change for each hospitalisation. Exposure to antimicrobials was assessed cumulatively until current hospitalisation.

*SHR and 95% CI for SHR obtained from the Fine-Gray model

**Days of exposure to different antibiotic classes were summed up irrespective if given in parallel or sequentially.

Table S9h: Variation with exposure to different antimicrobial groups in days (patient level)

	Univariate analysis			Multivariate analysis:		
**Variable**	**SHR***	**95% CI**	**p value**	**SHR***	**95% CI**	**p value**
Site group						
SCP						
NCP	1.71	1.35-2.17	<0.001	1.72	1.25-2.37	0.001
Age						
≤40 years						
41-60 years	1.28	0.87-1.88	0.215	1.29	0.87-1.92	0.209
>60 years	1.27	0.88-1.82	0.199	1.33	0.91-1.93	0.136
Sex						
Female						
Male	1.22	0.96-1.55	0.112	1.26	0.99-1.61	0.062
Underlying disease categorised						
Other						
Acute leukaemia	4.80	3.09-7.46	<0.001	3.57	2.24-5.67	<0.001
Lymphoma	2.39	1.55-3.70	<0.001	1.91	1.23-2.98	0.004
Solid tumor	1.25	0.77-2.04	0.368	1.15	0.70-1.87	0.584
Length of exposure to antimicrobials active against VRE in days#**	1.01	0.99-1.04	0.286	0.91	0.85-0.98	0.007
Length of exposure to cephalosporins in days#**	1.05	1.03-1.06	<0.001	1.03	1.00-1.07	0.030
Length of exposure to fluoroquinolones in days#**	1.05	1.03-1.06	<0.001	1.04	1.02-1.06	<0.001
Length of exposure to glycopeptides in days#**	1.04	1.03-1.06	<0.001	1.00	0.97-1.02	0.780
Length of exposure to other antimicrobials in days#**	1.02	1.01-1.03	<0.001	1.01	1.00-1.03	0.018
Compliance with hand hygiene at respective site during this hospitalisation#						
>75%						
≤75%	1.42	1.13-1.80	0.003	1.02	0.75-1.38	0.905

VRE: vancomycin-resistant enterococci; NCP: no contact precautions; SCP: single room contact precautions; SHR: subdistribution hazard ratio; CI: confidence interval

#Exposure to antimicrobials and compliance with hand hygiene were included as time-varying covariate and could change for each hospitalisation. Exposure to antimicrobials was assessed cumulatively until current hospitalisation.

*SHR and 95% CI for SHR obtained from the Fine-Gray model

**Days of exposure to different antibiotic classes were summed up irrespective if given in parallel or sequentially.

Table S9i: Variation with categorised length of exposure to different antimicrobial groups (patient level)

	Univariate analysis			Multivariate analysis:		
**Variable**	**SHR***	**95% CI**	**p value**	**SHR***	**95% CI**	**p value**
Site group						
SCP						
NCP	1.71	1.35-2.17	<0.001	1.66	1.18-2.35	0.004
Age						
≤40 years						
41-60 years	1.28	0.87-1.88	0.215	1.22	0.82-1.82	0.330
>60 years	1.27	0.88-1.82	0.199	1.32	0.91-1.93	0.142
Sex						
Female						
Male	1.22	0.96-1.55	0.112	1.26	0.99-1.61	0.061
Underlying disease categorised						
Other						
Acute leukaemia	4.80	3.09-7.46	<0.001	2.26	1.40-3.66	0.001
Lymphoma	2.39	1.55-3.70	<0.001	1.35	0.86-2.12	0.190
Solid tumor	1.25	0.77-2.04	0.368	1.20	0.74-1.96	0.458
Length of exposure to antimicrobials active against VRE in categories[#]**						
None						
≤7 days until now	1.47	0.75-2.88	0.260	0.74	0.37-1.49	0.395
>7 days until now	1.57	0.89-2.78	0.118	0.56	0.29-1.08	0.083
Length of exposure to cephalosporins in categories[#]**						
None						
≤7 days until now	3.45	2.56-4.64	<0.001	1.86	1.32-2.62	<0.001
>7 days until now	3.02	1.83-4.98	<0.001	1.47	0.83-2.61	0.187
Length of exposure to fluoroquinolones in categories[#]**						
None						
≤7 days until now	1.70	1.23-2.34	0.001	1.24	0.88-1.74	0.221
>7 days until now	3.30	2.43-4.47	<0.001	2.03	1.38-2.99	<0.001
Length of exposure to glycopeptides in categories[#]**						
None						
≤7 days until now	3.34	2.41-4.62	<0.001	1.70	1.19-2.42	0.004
>7 days until now	3.58	2.35-5.45	<0.001	1.28	0.80-2.05	0.307
Length of exposure to other antimicrobials in categories[#]**						
None						
≤7 days until now	5.93	3.94-8.92	<0.001	4.39	2.84-6.78	<0.001
>7 days until now	7.90	5.30-11.77	<0.001	4.27	2.67-6.82	<0.001
Compliance with hand hygiene at respective site during this hospitalisation[#]						
>75%						
≤75%	1.42	1.13-1.80	0.003	1.01	0.72-1.40	0.975

VRE: vancomycin-resistant enterococci; NCP: no contact precautions; SCP: single room contact precautions; SHR: subdistribution hazard ratio; CI: confidence interval

[#]Exposure to antimicrobials and compliance with hand hygiene were included as time-varying covariate and could change for each hospitalisation. Exposure to antimicrobials was assessed cumulatively until current hospitalisation.

*SHR and 95% CI for SHR obtained from the Fine-Gray model

**Days of exposure to different antibiotic classes were summed up irrespective if given in parallel or sequentially.

Tables S10a-j: Competing risk analysis for hospital-acquired VRE colonisation or bloodstream infection at the hospitalisation level: univariate and multivariate regression – Sensitivity analyses (variations highlighted in yellow)

Table S10a: Main model with exposure to different antimicrobial classes (hospitalisation level)

	**Univariate analysis**			**Multivariate analysis:**		
**Variable**	**SHR***	**95% CI**	**p value**	**SHR***	**95% CI**	**p value**
Site group						
SCP						
NCP	1.67	1.32-2.11	<0.001	1.60	1.13-2.27	0.009
Age						
≤40 years						
41-60 years	1.28	0.87-1.88	0.215	1.34	0.91-1.99	0.139
>60 years	1.27	0.88-1.82	0.198	1.44	1.00-2.08	0.052
Sex						
Female						
Male	1.22	0.96-1.55	0.104	1.28	1.01-1.63	0.043
Underlying disease categorised						
Other						
Acute leukaemia	3.17	2.04-4.91	<0.001	1.92	1.21-3.04	0.006
Lymphoma	1.84	1.19-2.85	0.006	1.15	0.73-1.80	0.544
Solid tumor	1.19	0.73-1.93	0.486	1.16	0.71-1.89	0.561
Exposure to antimicrobials active against VRE (lipopetides and oxazolidinones)						
No						
Yes	0.91	0.55-1.51	0.726	0.52	0.30-0.88	0.015
Exposure to cephalosporins						
No						
Yes	2.89	2.21-3.78	<0.001	1.80	1.31-2.47	<0.001
Exposure to fluoroquinolones						
No						
Yes	1.64	1.27-2.10	<0.001	1.27	0.93-1.72	0.132
Exposure to glycopeptides						
No						
Yes	2.48	1.89-3.26	<0.001	1.42	1.05-1.92	0.022
Exposure to other antimicrobials						
No						
Yes	4.72	3.32-6.73	<0.001	3.44	2.31-5.12	<0.001
Compliance with hand hygiene at respective site during this hospitalisation[#]						
>75%						
≤75%	1.35	1.07-1.71	0.010	0.97	0.70-1.34	0.865

VRE: vancomycin-resistant enterococci; NCP: no contact precautions; SCP: single room contact precautions; SHR: subdistribution hazard ratio; CI: confidence interval
*SHR and 95% CI for SHR obtained from the Fine-Gray model

Table S10b: Variation with separated sites (hospitalisation level)

	Univariate analysis			Multivariate analysis:		
**Variable**	**SHR***	**95% CI**	**p value**	**SHR***	**95% CI**	**p value**
Site						
SCP2						
SCP1	1.48	1.02-2.15	0.040	1.50	0.79-2.84	0.211
NCP1	2.45	1.76-3.41	<0.001	2.61	1.79-3.79	<0.001
NCP2	1.65	1.21-2.25	0.001	1.00	0.49-2.05	0.996
Age						
≤40 years						
41-60 years	1.28	0.87-1.88	0.215	1.37	0.93-2.02	0.110
>60 years	1.27	0.88-1.82	0.198	1.42	0.99-2.05	0.059
Sex						
Female						
Male	1.22	0.96-1.55	0.104	1.24	0.97-1.58	0.080
Underlying disease categorised						
Other						
Acute leukaemia	3.17	2.04-4.91	<0.001	1.64	1.03-2.61	0.036
Lymphoma	1.84	1.19-2.85	0.006	1.07	0.68-1.66	0.779
Solid tumor	1.19	0.73-1.93	0.486	1.06	0.65-1.75	0.803
Exposure to antimicrobials active against VRE (lipopetides and oxazolidinones)						
No						
Yes	0.91	0.55-1.51	0.726	0.55	0.32-0.93	0.027
Exposure to cephalosporins						
No						
Yes	2.89	2.21-3.78	<0.001	1.82	1.31-2.54	<0.001
Exposure to fluoroquinolones						
No						
Yes	1.64	1.27-2.10	<0.001	1.27	0.92-1.75	0.147
Exposure to glycopeptides						
No						
Yes	2.48	1.89-3.26	<0.001	1.29	0.95-1.77	0.107
Exposure to other antimicrobials						
No						
Yes	4.72	3.32-6.73	<0.001	3.87	2.47-6.07	<0.001
**Compliance with hand hygiene at respective site during this hospitalisation**[#]						
>75%						
≤75%	1.35	1.07-1.71	0.010	1.68	0.90-3.12	0.101

VRE: vancomycin-resistant enterococci; NCP: no contact precautions; SCP: single room contact precautions; SHR: subdistribution hazard ratio; CI: confidence interval

*SHR and 95% CI for SHR obtained from the Fine-Gray model

Table S10c: Variation with length of exposure to any antimicrobial class in days (hospitalisation level)

	**Univariate analysis**			**Multivariate analysis:**		
**Variable**	**SHR***	**95% CI**	**p value**	**SHR***	**95% CI**	**p value**
Site group						
SCP						
NCP	1.67	1.32-2.11	<0.001	1.72	1.26-2.36	0.001
Age						
≤40 years						
41-60 years	1.28	0.87-1.88	0.215	1.40	0.95-2.07	0.092
>60 years	1.27	0.88-1.82	0.198	1.43	0.99-2.06	0.057
Sex						
Female						
Male	1.22	0.96-1.55	0.104	1.28	1.01-1.63	0.045
Underlying disease categorised						
Other						
Acute leukaemia	3.17	2.04-4.91	<0.001	2.74	1.73-4.32	<0.001
Lymphoma	1.84	1.19-2.85	0.006	1.46	0.94-2.28	0.094
Solid tumor	1.19	0.73-1.93	0.486	1.05	0.65-1.72	0.834
Length of exposure to any antimicrobial in days**	1.01	1.00-1.01	<0.001	1.005	1.001-1.009	0.014
Compliance with hand hygiene at respective site during this hospitalisation[#]						
>75%						
≤75%	1.35	1.07-1.71	0.010	1.09	0.80-1.47	0.581

VRE: vancomycin-resistant enterococci; NCP: no contact precautions; SCP: single room contact precautions; SHR: subdistribution hazard ratio; CI: confidence interval

*SHR and 95% CI for SHR obtained from the Fine-Gray model

**Days of exposure to different antibiotic classes were summed up irrespective if given in parallel or sequentially.

Table S10d: Variation with exposure to any antimicrobial irrespective of length (hospitalisation level)

	**Univariate analysis**			**Multivariate analysis:**		
**Variable**	**SHR***	**95% CI**	**p value**	**SHR***	**95% CI**	**p value**
Site group						
SCP						
NCP	1.67	1.32-2.11	<0.001	1.36	0.99-1.87	0.060
Age						
≤40 years						
41-60 years	1.28	0.87-1.88	0.215	1.38	0.93-2.04	0.107
>60 years	1.27	0.88-1.82	0.198	1.39	0.97-2.01	0.077
Sex						
Female						
Male	1.22	0.96-1.55	0.104	1.31	1.03-1.66	0.030
Underlying disease categorised						
Other						
Acute leukaemia	3.17	2.04-4.91	<0.001	2.22	1.43-3.45	<0.001
Lymphoma	1.84	1.19-2.85	0.006	1.17	0.75-1.82	0.494
Solid tumor	1.19	0.73-1.93	0.486	1.21	0.74-1.97	0.456
Exposure to any antimicrobial						
No						
Yes	6.25	4.09-9.57	<0.001	5.46	3.48-8.58	<0.001
Compliance with hand hygiene at respective site during this hospitalisation[#]						
>75%						
≤75%	1.35	1.07-1.71	0.010	1.22	0.90-1.66	0.206

VRE: vancomycin-resistant enterococci; NCP: no contact precautions; SCP: single room contact precautions; SHR: subdistribution hazard ratio; CI: confidence interval

*SHR and 95% CI for SHR obtained from the Fine-Gray model

Table S10e: Variation with categorised length of exposure to any antimicrobial (hospitalisation level)

	Univariate analysis			Multivariate analysis:		
**Variable**	**SHR***	**95% CI**	**p value**	**SHR***	**95% CI**	**p value**
Site group						
SCP						
NCP	1.67	1.32-2.11	<0.001	1.40	1.02-1.93	0.038
Age						
≤40 years						
41-60 years	1.28	0.87-1.88	0.215	1.37	0.93-2.03	0.114
>60 years	1.27	0.88-1.82	0.198	1.41	0.98-2.04	0.068
Sex						
Female						
Male	1.22	0.96-1.55	0.104	1.30	1.02-1.66	0.032
Underlying disease categorised						
Other						
Acute leukaemia	3.17	2.04-4.91	<0.001	2.04	1.29-3.22	0.002
Lymphoma	1.84	1.19-2.85	0.006	1.11	0.71-1.75	0.637
Solid tumor	1.19	0.73-1.93	0.486	1.17	0.71-1.91	0.534
Exposure to any antimicrobial**						
None						
≤7 days	4.84	3.04-7.70	<0.001	4.50	2.79-7.27	<0.001
>7 days	7.18	4.66-11.04	<0.001	6.11	3.84-9.72	<0.001
Compliance with hand hygiene at respective site during this hospitalisation[#]						
>75%						
≤75%	1.35	1.07-1.71	0.010	1.20	0.88-1.63	0.242

VRE: vancomycin-resistant enterococci; NCP: no contact precautions; SCP: single room contact precautions; SHR: subdistribution hazard ratio; CI: confidence interval

*SHR and 95% CI for SHR obtained from the Fine-Gray model

**Days of exposure to different antibiotic classes were summed up irrespective if given in parallel or sequentially.

Table S10f: Variation with length of exposure to antimicrobials active or not active against VRE in days (hospitalisation level)

	Univariate analysis			Multivariate analysis:		
**Variable**	**SHR***	**95% CI**	**p value**	**SHR***	**95% CI**	**p value**
Site group						
SCP						
NCP	1.67	1.32-2.11	<0.001	1.67	1.22-2.27	0.001
Age						
≤40 years						
41-60 years	1.28	0.87-1.88	0.215	1.40	0.94-2.07	0.094
>60 years	1.27	0.88-1.82	0.198	1.47	1.02-2.13	0.039
Sex						
Female						
Male	1.22	0.96-1.55	0.104	1.28	1.00-1.63	0.046
Underlying disease categorised						
Other						
Acute leukaemia	3.17	2.04-4.91	<0.001	2.67	1.71-4.17	<0.001
Lymphoma	1.84	1.19-2.85	0.006	1.49	0.96-2.31	0.078
Solid tumor	1.19	0.73-1.93	0.486	1.08	0.66-1.75	0.771
Length of exposure to antimicrobials active against VRE in days (lipopeptides and oxazolidinones)**	0.97	0.93-1.02	0.239	0.93	0.89-0.98	0.004
Length of exposure to antimicrobials not active against VRE in days**	1.01	1.006-1.014	<0.001	1.013	1.008-1.018	<0.001
Compliance with hand hygiene at respective site during this hospitalisation[#]						
>75%						
≤75%	1.35	1.07-1.71	0.010	1.04	0.77-1.40	0.812

VRE: vancomycin-resistant enterococci; NCP: no contact precautions; SCP: single room contact precautions; SHR: subdistribution hazard ratio; CI: confidence interval
*SHR and 95% CI for SHR obtained from the Fine-Gray model
**Days of exposure to different antibiotic classes were summed up irrespective if given in parallel or sequentially.

Table S10g: Variation with exposure to antimicrobials active or not active against VRE irrespective of length (hospitalisation level)

	Univariate analysis			Multivariate analysis:		
**Variable**	**SHR***	**95% CI**	**p value**	**SHR***	**95% CI**	**p value**
Site group						
SCP						
NCP	1.67	1.32-2.11	<0.001	1.32	0.95-1.82	0.095
Age						
≤40 years						
41-60 years	1.28	0.87-1.88	0.215	1.39	0.94-2.06	0.098
>60 years	1.27	0.88-1.82	0.198	1.40	0.97-2.01	0.074
Sex						
Female						
Male	1.22	0.96-1.55	0.104	1.30	1.02-1.65	0.032
Underlying disease categorised						
Other						
Acute leukaemia	3.17	2.04-4.91	<0.001	2.35	1.50-3.66	<0.001
Lymphoma	1.84	1.19-2.85	0.006	1.20	0.77-1.88	0.422
Solid tumor	1.19	0.73-1.93	0.486	1.21	0.74-1.99	0.438
Exposure to antimicrobials active against VRE (lipopetides and oxazolidinones)						
No						
Yes	0.91	0.55-1.51	0.726	0.60	0.35-1.01	0.053
Exposure to Non-antimicrobials not active against VRE						
No						
Yes	6.25	4.09-9.57	<0.001	5.65	3.60-8.87	<0.001
Compliance with hand hygiene at respective site during this hospitalisation[#]						
>75%						
≤75%	1.35	1.07-1.71	0.010	1.17	0.86-1.59	0.308

VRE: vancomycin-resistant enterococci; NCP: no contact precautions; SCP: single room contact precautions; SHR: subdistribution hazard ratio; CI: confidence interval
*SHR and 95% CI for SHR obtained from the Fine-Gray model

Table S10h: Variation with categorised length of exposure to antimicrobials active or not active against VRE (hospitalisation level)

	Univariate analysis			Multivariate analysis:		
**Variable**	**SHR***	**95% CI**	**p value**	**SHR***	**95% CI**	**p value**
Site group						
SCP						
NCP	1.67	1.32-2.11	<0.001	1.34	0.97-1.85	0.072
Age						
≤40 years						
41-60 years	1.28	0.87-1.88	0.215	1.38	0.94-2.05	0.104
>60 years	1.27	0.88-1.82	0.198	1.41	0.98-2.04	0.065
Sex						
Female						
Male	1.22	0.96-1.55	0.104	1.30	1.02-1.65	0.033
Underlying disease categorised						
Other						
Acute leukaemia	3.17	2.04-4.91	<0.001	2.16	1.37-3.42	0.001
Lymphoma	1.84	1.19-2.85	0.006	1.15	0.73-1.81	0.541
Solid tumor	1.19	0.73-1.93	0.486	1.18	0.72-1.93	0.516
Length of exposure to antimicrobials active against VRE in categories (lipopetides and oxazolidinones)**						
None						
≤7 days	1.13	0.58-2.18	0.720	0.69	0.35-1.35	0.282
>7 days	0.73	0.35-1.55	0.418	0.43	0.20-0.92	0.030
Length of exposure to antimicrobials not active against VRE antimicrobials in categories**						
None						
≤7 days	4.88	3.07-7.76	<0.001	4.58	2.84-7.39	<0.001
>7 days	7.18	4.66-11.05	<0.001	6.46	4.07-10.27	<0.001
Compliance with hand hygiene at respective site during this hospitalisation[#]						
>75%						
≤75%	1.35	1.07-1.71	0.010	1.14	0.84-1.55	0.389

VRE: vancomycin-resistant enterococci; NCP: no contact precautions; SCP: single room contact precautions; SHR: subdistribution hazard ratio; CI: confidence interval

*SHR and 95% CI for SHR obtained from the Fine-Gray model

**Days of exposure to different antibiotic classes were summed up irrespective if given in parallel or sequentially.

Table S10i: Variation with length of exposure to antimicrobial class in days (hospitalisation level)

	Univariate analysis			Multivariate analysis:		
**Variable**	**SHR***	**95% CI**	**p value**	**SHR***	**95% CI**	**p value**
Site group						
SCP						
NCP	1.67	1.32-2.11	<0.001	1.87	1.37-2.57	<0.001
Age						
≤40 years						
41-60 years	1.28	0.87-1.88	0.215	1.44	0.97-2.13	0.068
>60 years	1.27	0.88-1.82	0.198	1.46	1.01-2.11	0.044
Sex						
Female						
Male	1.22	0.96-1.55	0.104	1.32	1.04-1.68	0.02
Underlying disease categorised						
Other						
Acute leukaemia	3.17	2.04-4.91	<0.001	2.84	1.80-4.48	<0.001
Lymphoma	1.84	1.19-2.85	0.006	1.56	1.00-2.44	0.050
Solid tumor	1.19	0.73-1.93	0.486	1.10	0.67-1.80	0.706
Length of exposure to antimicrobials active against VRE in days (lipopetides and oxazolidinones) **	0.97	0.93-1.02	0.239	0.90	0.84-0.97	0.003
Length of exposure to cephalosporins in days**	1.04	1.03-1.06	<0.001	1.06	1.03-1.09	<0.001
Length of exposure to fluoroquinolones in days**	1.03	1.02-1.05	<0.001	1.04	1.01-1.06	0.002
Length of exposure to glycopeptides in days**	1.03	1.02-1.04	<0.001	0.98	0.96-1.01	0.174
Length of other antimicrobials in days**	1.01	1.007-1.02	<0.001	1.00	0.99-1.01	0.694
Compliance with hand hygiene at respective site during this hospitalisation[#]						
>75%						
≤75%	1.35	1.07-1.71	0.010	0.97	0.72-1.30	0.828

VRE: vancomycin-resistant enterococci; SHR: subdistribution hazard ratio; CI: confidence interval
*SHR and 95% CI for SHR obtained from the Fine-Gray model
**Days of exposure to different antibiotic classes were summed up irrespective if given in parallel or sequentially.

Table S10j: Variation with categorised length of antimicrobial classes (hospitalisation level)

	Univariate analysis			Multivariate analysis:		
**Variable**	**SHR***	**95% CI**	**p value**	**SHR***	**95% CI**	**p value**
Site group						
SCP						
NCP	1.67	1.32-2.11	<0.001	1.69	1.20-2.38	0.003
Age						
≤40 years						
41-60 years	1.28	0.87-1.88	0.215	1.36	0.92-2.00	0.123
>60 years	1.27	0.88-1.82	0.198	1.45	1.01-2.09	0.045
Sex						
Female						
Male	1.22	0.96-1.55	0.104	1.32	1.04-1.68	0.023
Underlying disease categorised						
Other						
Acute leukaemia	3.17	2.04-4.91	<0.001	1.95	1.22-3.12	0.005
Lymphoma	1.84	1.19-2.85	0.006	1.15	0.73-1.80	0.545
Solid tumor	1.19	0.73-1.93	0.486	1.15	0.70-1.87	0.589
Length of exposure to antimicrobials active against VRE in categories (lipopetides and oxazolidinones) **						
None						
≤7 days	1.13	0.58-2.18	0.720	0.73	0.37-1.44	0.368
>7 days	0.73	0.35-1.55	0.418	0.38	0.17-0.85	0.019
Length of exposure to cephalosporins in categories**						
None						
≤7 days	3.08	2.29-4.13	<0.001	2.06	1.47-2.90	<0.001
>7 days	2.37	1.41-3.97	0.001	1.64	0.92-2.90	0.091
Length of exposure to fluoroquinolones in categories**						
None						
≤7 days	1.32	0.94-1.85	0.109	1.08	0.75-1.57	0.666
>7 days	2.10	1.53-2.88	<0.001	1.69	1.12-2.54	0.012
Length of exposure to glycopeptides in categories**						
None						
≤7 days	2.75	2.01-3.77	<0.001	1.78	1.27-2.51	0.001
>7 days	1.94	1.21-3.11	0.006	0.93	0.57-1.53	0.780
Length of other antimicrobials**						
None						
≤7 days	4.98	3.42-7.23	<0.001	3.79	2.53-5.68	<0.001
>7 days	4.47	3.08-6.49	<0.001	2.80	1.80-4.36	<0.001
Compliance with hand hygiene at respective site during this hospitalisation[#]						
>75%						
≤75%	1.35	1.07-1.71	0.010	0.94	0.68-1.28	0.684

VRE: vancomycin-resistant enterococci; NCP: no contact precautions; SCP: single room contact precautions; SHR: subdistribution hazard ratio; CI: confidence interval

*SHR and 95% CI for SHR obtained from the Fine-Gray model

**Days of exposure to different antibiotic classes were summed up irrespective if given in parallel or sequentially.

Tables S11a-k: Competing risk analysis for confirmed patient-to-patient transmission of VRE at the patient level: univariate and multivariate regression – (variations highlighted in yellow)

Table S11a: Main model (patient level)

	**Univariate analysis**			**Multivariate analysis****		
**Variable**	**SHR***	**95% CI**	**p value**	**SHR***	**95% CI**	**p value**
Site group						
SCP						
NCP	1.73	1.32-2.28	<0.001	1.62	1.09-2.41	0.018
Age in categories						
≤40 years						
41 to 60 years	1.35	0.87-2.11	0.182	1.39	0.88-2.18	0.156
>60 years	1.15	0.75-1.75	0.526	1.29	0.84-1.98	0.239
Sex						
Female						
Male	1.13	0.86-1.48	0.398	1.17	0.89-1.54	0.267
Underlying haematological disease in categories						
Solid tumor	1.27	0.74-2.19	0.387	1.23	0.71-2.12	0.464
Acute leukaemia	4.93	3.01-8.05	0.000	2.35	1.39-3.97	0.001
Lymphoma	1.97	1.20-3.24	0.007	1.09	0.65-1.81	0.747
Other						
Exposure to antimicrobials active against VRE- #						
No						
Yes	1.40	0.83-2.35	0.202	0.62	0.35-1.01	0.103
Exposure to cephalosporins#						
No						
Yes	3.27	2.41-4.45	<0.001	1.54	1.07-2.22	0.021
Exposure to fluoroquinolones#						
No						
Yes	2.46	1.87-3.24	<0.001	1.66	1.21-2.26	0.001
Exposure to glycopeptides#						
No						
Yes	3.51	2.58-4.78	<0.001	1.58	1.11-2.25	0.011
Exposure to other antimicrobials#						
No						
Yes	7.08	4.51-11.11	<0.001	4.67	2.83-7.69	<0.001
Compliance with hand hygiene at respective site during this hospitalisation#						
>75%						
≤75%	1.56	1.19-2.03	0.001	1.14	0.77-1.69	0.503

VRE: vancomycin-resistant enterococci*;* NCP: no contact precautions; SCP: single room contact precautions;
SHR: subdistribution hazard ratio; CI: confidence interval
#Exposure to antimicrobials and compliance with hand hygiene were included as time-varying covariate and could change for each hospitalisation. Exposure to antimicrobials was assessed cumulatively until current hospitalisation.
*SHR and 95% CI for SHR obtained from the Fine-Gray model;
**Adjusted multivariable model for site, age, sex, underlying disease, exposure to different antimicrobial classes; hand hygiene compliance.

Table S11b: Variation with separate sites (patient level)

	Univariate analysis			Multivariate analysis:		
**Variable**	**SHR***	**95% CI**	**p value**	**SHR***	**95% CI**	**p value**
Site						
SCP2						
SCP1	1.76	1.14-2.70	0.010	1.45	0.63-3.33	0.380
NCP1	2.73	1.85-4.01	<0.001	3.03	1.99-4.61	<0.001
NCP2	1.83	1.28-2.61	0.001	0.86	0.34-2.16	0.745
Age						
≤40 years						
41-60 years	1.35	0.87-2.11	0.182	1.39	0.89-2.18	0.150
>60 years	1.15	0.75-1.75	0.526	1.26	0.82-1.94	0.282
Sex						
Female						
Male	1.13	0.86-1.48	0.398	1.13	0.86-1.50	0.380
Underlying disease categorised						
Other						
Acute leukaemia	4.93	3.01-8.05	<0.001	1.96	1.16-3.31	0.012
Lymphoma	1.97	1.20-3.24	0.007	1.00	0.60	1.65
Solid tumor	1.27	0.74-2.19	0.387	1.14	0.66-1.97	0.646
Exposure to antimicrobials active against VRE#						
No						
Yes	1.40	0.83-2.35	0.202	0.74	0.41-1.30	0.293
Exposure to cephalosporins#						
No						
Yes	3.27	2.41-4.45	<0.001	1.59	1.08-2.34	0.020
Exposure to fluoroquinolones#						
No						
Yes	2.46	1.87-3.24	<0.001	1.64	1.18-2.27	0.003
Exposure to glycopeptides#						
No						
Yes	3.51	2.58-4.78	<0.001	1.41	0.97-2.04	0.074
Exposure to other antimicrobials#						
No						
Yes	7.08	4.51-11.11	<0.001	5.14	2.98-8.88	<0.001
Compliance with hand hygiene at respective site during this hospitalisation#						
>75%						
≤75%	1.56	1.19-2.03	0.001	2.40	1.04-5.51	0.039

VRE: vancomycin-resistant enterococci; NCP: no contact precautions; SCP: single room contact precautions; SHR: subdistribution hazard ratio; CI: confidence interval

#Exposure to antimicrobials and compliance with hand hygiene were included as time-varying covariate and could change for each hospitalisation. Exposure to antimicrobials was assessed cumulatively until current hospitalisation.

*SHR and 95% CI for SHR obtained from the Fine-Gray model.

Table S11c: Variation with exposure to any antimicrobial in days (patient level)

	Univariate analysis			Multivariate analysis:		
**Variable**	**SHR***	**95% CI**	**p value**	**SHR***	**95% CI**	**p value**
Site group						
SCP						
NCP	1.73	1.32-2.28	<0.001	1.62	1.10-2.39	0.014
Age						
≤40 years						
41-60 years	1.35	0.87-2.11	0.182	1.46	0.94-2.29	0.095
>60 years	1.15	0.75-1.75	0.526	1.28	0.83-1.97	0.257
Sex						
Female						
Male	1.13	0.86-1.48	0.398	1.17	0.88-1.54	0.276
Underlying disease categorised						
Other						
Acute leukaemia	4.93	3.01-8.05	<0.001	3.86	2.32-6.43	<0.001
Lymphoma	1.97	1.20-3.24	0.007	1.54	0.93-2.55	0.093
Solid tumor	1.27	0.74-2.19	0.387	1.13	0.65-1.95	0.665
Length of exposure to any antimicrobial in days[#]**	1.01	1.006-1.015	<0.001	1.01	1.003-1.012	0.001
Compliance with hand hygiene at respective site during this hospitalisation[#]						
>75%						
≤75%	1.56	1.19-2.03	0.001	1.25	0.86-1.81	0.240

VRE: vancomycin-resistant enterococci; NCP: no contact precautions; SCP: single room contact precautions; SHR: subdistribution hazard ratio; CI: confidence interval

[#]Exposure to antimicrobials and compliance with hand hygiene were included as time-varying covariate and could change for each hospitalisation. Exposure to antimicrobials was assessed cumulatively until current hospitalisation.

*SHR and 95% CI for SHR obtained from the Fine-Gray model
**Days of exposure to different antibiotic classes were summed up irrespective if given in parallel or sequentially.

Table S11d: Variation with exposure to any antimicrobial in days and sites separately (patient level)

	Univariate analysis			Multivariate analysis:		
**Variable**	**SHR***	**95% CI**	**p value**	**SHR***	**95% CI**	**p value**
Site						
SCP2						
SCP1	1.76	1.14-2.70	0.010	0.50	0.21-1.18	0.115
NCP1	2.73	1.85-4.01	<0.001	2.70	1.80-4.05	<0.001
NCP2	1.83	1.28-2.61	0.001	0.31	0.12-0.81	0.016
Age						
≤40 years						
41-60 years	1.35	0.87-2.11	0.182	1.44	0.92-2.25	0.110
>60 years	1.15	0.75-1.75	0.526	1.25	0.81-1.92	0.313
Sex						
Female						
Male	1.13	0.86-1.48	0.398	1.16	0.88-1.54	0.292
Underlying disease categorised						
Other						
Acute leukaemia	4.93	3.01-8.05	<0.001	3.38	2.02-5.64	<0.001
Lymphoma	1.97	1.20-3.24	0.007	1.41	0.85-2.34	0.180
Solid tumor	1.27	0.74-2.19	0.387	1.03	0.59-1.80	0.914
Length of exposure to any antimicrobial in days#**	1.01	1.006-1.015	<0.001	1.01	1.003-1.012	0.001
Compliance with hand hygiene at respective site during this hospitalisation#						
>75%						
≤75%	1.56	1.19-2.03	0.001	6.34	2.66-15.10	<0.001

VRE: vancomycin-resistant enterococci; NCP: no contact precautions; SCP: single room contact precautions; SHR: subdistribution hazard ratio; CI: confidence interval

#Exposure to antimicrobials and compliance with hand hygiene were included as time-varying covariate and could change for each hospitalisation. Exposure to antimicrobials was assessed cumulatively until current hospitalisation.

*SHR and 95% CI for SHR obtained from the Fine-Gray model

**Days of exposure to different antibiotic classes were summed up irrespective if given in parallel or sequentially.

Table S11e: Variation with exposure to any antimicrobial irrespective of length (patient level)

	**Univariate analysis**			**Multivariate analysis:**		
**Variable**	**SHR***	**95% CI**	**p value**	**SHR***	**95% CI**	**p value**
Site group						
SCP						
NCP	1.73	1.32-2.28	<0.001	1.23	0.83-1.83	0.293
Age						
≤40 years						
41-60 years	1.35	0.87-2.11	0.182	1.44	0.92-2.25	0.113
>60 years	1.15	0.75-1.75	0.526	1.26	0.82-1.93	0.296
Sex						
Female						
Male	1.13	0.86-1.48	0.398	1.19	0.90-1.57	0.215
Underlying disease categorised						
Other						
Acute leukaemia	4.93	3.01-8.05	<0.001	3.14	1.91-5.16	<0.001
Lymphoma	1.97	1.20-3.24	0.007	1.20	0.72-1.98	0.490
Solid tumor	1.27	0.74-2.19	0.387	1.33	0.77-2.30	0.311
Exposure to any antimicrobial[#]						
No						
Yes	9.95	5.67-17.43	<0.001	8.45	4.72-15.14	<0.001
Compliance with hand hygiene at respective site during this hospitalisation[#]						
>75%						
≤75%	1.56	1.19-2.03	0.001	1.41	0.97-2.06	0.074

VRE: vancomycin-resistant enterococci; NCP: no contact precautions; SCP: single room contact precautions; SHR: subdistribution hazard ratio; CI: confidence interval

[#]Exposure to antimicrobials and compliance with hand hygiene were included as time-varying covariate and could change for each hospitalisation. Exposure to antimicrobials was assessed cumulatively until current hospitalisation.

*SHR and 95% CI for SHR obtained from the Fine-Gray model

Table S11f: Variation with length of exposure to any antimicrobials in categories (patient level)

	Univariate analysis			Multivariate analysis:		
**Variable**	**SHR***	**95% CI**	**p value**	**SHR***	**95% CI**	**p value**
Site group						
SCP						
NCP	1.73	1.32-2.28	<0.001	1.29	0.87-1.90	0.204
Age						
≤40 years						
41-60 years	1.35	0.87-2.11	1.182	1.42	0.90-2.22	0.129
>60 years	1.15	0.75-1.75	0.526	1.27	0.82-1.94	0.282
Sex						
Female						
Male	1.13	0.86-1.48	0.398	1.18	0.89-1.56	0.241
Underlying disease categorised						
Other						
Acute leukaemia	4.93	3.01-8.05	<0.001	2.67	1.60-4.47	<0.001
Lymphoma	1.97	1.20-3.24	0.007	1.11	0.67-1.85	0.684
Solid tumor	1.27	0.74-2.19	0.387	1.25	0.72-2.17	0.417
Length of exposure to any antimicrobial in categories (different antibiotic classes cumulated) #**						
None						
≤7 days until now	6.40	3.50-11.71	<0.001	6.18	3.34-11.42	<0.001
>7 days until now	12.70	7.21-22.37	<0.001	10.21	5.60-18.58	<0.001
Compliance with hand hygiene at respective site during this hospitalisation#						
>75%						
≤75%	1.56	1.19-2.03	0.001	1.38	0.95-2.01	0.095

VRE: vancomycin-resistant enterococci; NCP: no contact precautions; SCP: single room contact precautions; SHR: subdistribution hazard ratio; CI: confidence interval

#Exposure to antimicrobials and compliance with hand hygiene were included as time-varying covariate and could change for each hospitalisation. Exposure to antimicrobials was assessed cumulatively until current hospitalisation.

*SHR and 95% CI for SHR obtained from the Fine-Gray model

**Days of exposure to different antibiotic classes were summed up irrespective if given in parallel or sequentially.

Table S11g: Variation with length of exposure to antimicrobials active and not active against VRE in days (patient level)

	Univariate analysis			Multivariate analysis:		
**Variable**	**SHR***	**95% CI**	**p value**	**SHR***	**95% CI**	**p value**
Site group						
SCP						
NCP	1.73	1.32-2.28	<0.001	1.58	1.08-2.31	0.019
Age						
≤40 years						
41-60 years	1.35	0.87-2.11	0.182	1.46	0.93-2.30	0.098
>60 years	1.15	0.75-1.75	0.526	1.34	0.87-2.07	0.189
Sex						
Female						
Male	1.13	0.86-1.48	0.398	1.16	0.88-1.54	0.288
Underlying disease categorised						
Other						
Acute leukaemia	4.93	3.01-8.05	<0.001	3.74	2.25-6.24	<0.001
Lymphoma	1.97	1.20-3.24	0.007	1.56	0.94-2.59	0.083
Solid tumor	1.27	0.74-2.19	0.387	1.15	0.66-1.99	0.622
Length of exposure to antimicrobials active against VRE in days (lipopeptides and oxazolidinones) #**	1.01	0.98-1.04	0.597	0.95	0.90-0.99	0.025
Length of exposure to antimicrobials not active against VRE in days#**	1.01	1.01-1.02	<0.001	1.01	1.01-1.02	<0.001
Compliance with hand hygiene at respective site during this hospitalisation#						
>75%						
≤75%	1.56	1.19-2.03	0.001	1.17	0.81-1.69	0.394

VRE: vancomycin-resistant enterococci; NCP: no contact precautions; SCP: single room contact precautions; SHR: subdistribution hazard ratio; CI: confidence interval

#Exposure to antimicrobials and compliance with hand hygiene were included as time-varying covariate and could change for each hospitalisation. Exposure to antimicrobials was assessed cumulatively until current hospitalisation.

*SHR and 95% CI for SHR obtained from the Fine-Gray model

**Days of exposure to different antibiotic classes were summed up irrespective if given in parallel or sequentially.

Table S11h: Variation with exposure to antimicrobials active and not active against VRE irrespective of length (patient level)

	Univariate analysis			Multivariate analysis:		
**Variable**	**SHR***	**95% CI**	**p value**	**SHR***	**95% CI**	**p value**
Site group						
SCP						
NCP	1.73	1.32-2.28	<0.001	1.21	0.82-1.80	0.333
Age						
≤40 years						
41-60 years	1.35	0.87-2.11	0.182	1.44	0.92-2.27	0.110
>60 years	1.15	0.75-1.75	0.526	1.26	0.82-1.93	0.293
Sex						
Female						
Male	1.13	0.86-1.48	0.398	1.19	0.91-1.57	0.210
Underlying disease categorised						
Other						
Acute leukaemia	4.93	3.01-8.05	<0.001	3.25	1.96-5.39	<0.001
Lymphoma	1.97	1.20-3.24	0.007	1.21	0.73-2.02	0.453
Solid tumor	1.27	0.74-2.19	0.387	1.34	0.77-2.31	0.301
Exposure to antimicrobials active against VRE (lipopetides and oxazolidinones) #						
No						
Yes	1.40	0.83-2.35	2.202	0.77	0.44-1.37	0.378
Exposure to Non-antimicrobials not active against VRE#						
No						
Yes	9.95	5.67-17.43	<0.001	8.58	4.79-15.37	<0.001
Compliance with hand hygiene at respective site during this hospitalisation#						
>75%						
≤75%	1.56	1.19-2.03	0.001	1.37	0.94-2.00	0.098

VRE: vancomycin-resistant enterococci; NCP: no contact precautions; SCP: single room contact precautions; SHR: subdistribution hazard ratio; CI: confidence interval

#Exposure to antimicrobials and compliance with hand hygiene were included as time-varying covariate and could change for each hospitalisation. Exposure to antimicrobials was assessed cumulatively until current hospitalisation.

*SHR and 95% CI for SHR obtained from the Fine-Gray model

Table S11i: Variation with categorised length of exposure to antimicrobials active and not active against VRE (patient level)

	Univariate analysis			Multivariate analysis:		
**Variable**	**SHR***	**95% CI**	**p value**	**SHR***	**95% CI**	**p value**
Site group						
SCP						
NCP	1.73	1.32-2.28	<0.001	1.25	0.85-1.85	0.256
Age						
≤40 years						
41-60 years	1.35	0.87-2.11	0.182	1.43	0.91-2.24	0.122
>60 years	1.15	0.75-1.75	0.526	1.27	0.83-1.95	0.277
Sex						
Female						
Male	1.13	0.86-1.48	0.398	1.19	0.90-1.57	0.227
Underlying disease categorised						
Other						
Acute leukaemia	4.93	3.01-8.05	<0.001	2.81	1.68-4.72	<0.001
Lymphoma	1.97	1.20-3.24	0.007	1.14	0.68-1.89	0.620
Solid tumor	1.27	0.74-2.19	0.387	1.26	0.73-2.19	0.403
Length of exposure to antimicrobials active against VRE in categories (lipopeptides and oxazolidinones) [#]**						
None						
≤7 days until now	1.47	0.69-3.12	0.320	0.79	0.36-1.72	0.548
>7 days until now	1.35	0.67-2.71	0.399	0.59	0.28-1.24	0.167
Length of exposure to antimicrobials not active against VRE in categories[#]**						
None						
≤7 days until now	6.42	3.51-11.74	<0.001	6.21	3.37-11.45	<0.001
>7 days until now	12.75	7.24-22.46	<0.001	10.61	5.83-19.29	<0.001
Compliance with hand hygiene at respective site during this hospitalisation[#]						
>75%						
≤75%	1.56	1.19-2.03	0.001	1.31	0.90-1.91	0.153

VRE: vancomycin-resistant enterococci; NCP: no contact precautions; SCP: single room contact precautions; SHR: subdistribution hazard ratio; CI: confidence interval

[#]Exposure to antimicrobials and compliance with hand hygiene were included as time-varying covariate and could change for each hospitalisation. Exposure to antimicrobials was assessed cumulatively until current hospitalisation.

*SHR and 95% CI for SHR obtained from the Fine-Gray model

**Days of exposure to different antibiotic classes were summed up irrespective if given in parallel or sequentially.

Table S11j: Variation with exposure to different antimicrobial groups in days (patient level)

	**Univariate analysis**			**Multivariate analysis:**		
**Variable**	SHR*	95% CI	p value	SHR*	95% CI	p value
Site group						
SCP						
NCP	1.73	1.32-2.28	<0.001	1.70	1.16-2.50	0.006
Age						
≤40 years						
41-60 years	1.35	0.87-2.11	0.182	1.46	0.93-2.29	0.101
>60 years	1.15	0.75-1.75	0.526	1.30	0.84-2.00	0.235
Sex						
Female						
Male	1.13	0.86-1.48	0.398	1.20	0.91-1.58	0.199
Underlying disease categorised						
Other						
Acute leukaemia	4.93	3.01-8.05	<0.001	3.60	2.15-6.05	<0.001
Lymphoma	1.97	1.20-3.24	0.007	1.52	0.92-2.51	0.104
Solid tumor	1.27	0.74-2.19	0.387	1.13	0.65-1.95	0.668
Length of exposure to exposure to Antimicrobials active against VRE in days#**	1.01	0.98-1.04	0.597	0.92	0.86-0.98	0.010
Length of exposure to cephalosporins in days#**	1.04	1.02-1.06	<0.001	1.02	0.98-1.06	0.360
Length of exposure to fluoroquinolones in days#**	1.05	1.03-1.06	<0.001	1.05	1.02-1.07	<0.001
Length of exposure to glycopeptides in days#**	1.04	1.02-1.06	<0.001	0.99	0.96-1.02	0.448
Length of exposure to other antimicrobials in days#**	1.02	1.01-1.03	<0.001	1.01	1.00-1.03	0.028
Compliance with hand hygiene at respective site during this hospitalisation#						
>75%						
≤75%	1.56	1.19-2.03	0.001	1.15	0.80-1.66	0.460

VRE: vancomycin-resistant enterococci; NCP: no contact precautions; SCP: single room contact precautions; SHR: subdistribution hazard ratio; CI: confidence interval

#Exposure to antimicrobials and compliance with hand hygiene were included as time-varying covariate and could change for each hospitalisation. Exposure to antimicrobials was assessed cumulatively until current hospitalisation.

*SHR and 95% CI for SHR obtained from the Fine-Gray model

**Days of exposure to different antibiotic classes were summed up irrespective if given in parallel or sequentially.

Table S11k: Variation with categorised length of exposure to different antimicrobial groups (patient level)

	Univariate analysis			Multivariate analysis:		
**Variable**	**SHR***	**95% CI**	**p value**	**SHR***	**95% CI**	**p value**
Site group						
SCP						
NCP	1.73	1.32-2.28	<0.001	1.71	1.15-2.55	0.009
Age						
≤40 years						
41-60 years	1.35	0.87-2.11	0.182	1.38	0.87-2.17	0.167
>60 years	1.15	0.75-1.75	0.526	1.29	0.84-1.99	0.241
Sex						
Female						
Male	1.13	0.86-1.48	0.398	1.18	0.90-1.56	0.232
Underlying disease categorised						
Other						
Acute leukaemia	4.93	3.01-8.05	<0.001	2.29	1.35-3.91	0.002
Lymphoma	1.97	1.20-3.24	0.007	1.08	0.65-1.80	0.759
Solid tumor	1.27	0.74-2.19	0.387	1.21	0.70-2.10	0.487
Length of exposure to exposure to antimicrobials active against VRE in categories#**						
None						
≤7 days until now	1.47	0.69-3.12	0.320	0.76	0.35-1.67	0.497
>7 days until now	1.35	0.67-2.71	0.399	0.49	0.23-1.05	0.066
Length of exposure to cephalosporins in categories#**						
None						
≤7 days until now	3.57	2.56-4.97	<0.001	1.75	1.19-2.58	0.005
>7 days until now	2.42	1.28-4.57	0.007	1.07	0.52-2.18	0.853
Length of exposure to fluoroquinolones in categories#**						
None						
≤7 days until now	1.81	1.25-2.60	0.001	1.37	0.94-2.00	0.104
>7 days until now	3.61	2.57-5.09	<0.001	2.30	1.50-3.52	<0.001
Length of exposure to glycopeptides in categories#**						
None						
≤7 days until now	3.36	2.32-4.87	<0.001	1.66	1.09-2.52	0.018
>7 days until now	3.84	2.40-6.13	<0.001	1.37	0.81-2.32	0.242
Length of exposure to other antimicrobials in categories#**						
None						
≤7 days until now	6.38	3.96-10.27	<0.001	4.86	2.94-8.05	<0.001
>7 days until now	7.87	4.94-12.56	<0.001	4.32	2.49-7.49	<0.001
Compliance with hand hygiene at respective site during this hospitalisation#						
>75%						
≤75%	1.56	1.19-2.03	0.001	1.10	0.75-1.62	0.618

VRE: vancomycin-resistant enterococci; NCP: no contact precautions; SCP: single room contact precautions; SHR: subdistribution hazard ratio; CI: confidence interval

#Exposure to antimicrobials and compliance with hand hygiene were included as time-varying covariate and could change for each hospitalisation. Exposure to antimicrobials was assessed cumulatively until current hospitalisation.

*SHR and 95% CI for SHR obtained from the Fine-Gray model

**Days of exposure to different antibiotic classes were summed up irrespective if given in parallel or sequentially.

Tables S12a-k: Competing risk analysis for patient-to-patient transmission of at the hospitalisation level: univariate and multivariate regression – Sensitivity analyses (variations highlighted in yellow)

Table S121a: Main model with exposure to different antimicrobial classes (hospitalisation level)

	**Univariate analysis**			**Multivariate analysis:**		
**Variable**	**SHR***	**95% CI**	**p value**	**SHR***	**95% CI**	**p value**
Site group						
SCP						
NCP	1.73	1.32-2.28	<0.001	1.55	1.03-2.32	0.035
Age						
≤40 years						
41-60 years	1.35	0.87-2.11	0.182	1.39	0.89-2.19	0.149
>60 years	1.15	0.75-1.75	0.526	2.29	0.84-1.98	0.249
Sex						
Female						
Male	1.13	0.86-1.48	0.398	1.18	0.89-1.55	0.251
Underlying haematological disease categorised						
Other						
Acute leukaemia	4.93	3.01-8.05	<0.001	2.46	1.46-4.15	0.001
Lymphoma	1.97	1.20-3.24	0.007	1.13	0.68-1.89	0.627
Solid tumor	1.27	0.74-2.19	0.387	1.25	0.72-2.17	0.423
Exposure to antimicrobials active against VRE (lipopetides and oxazolidinones)						
No						
Yes	1.12	0.61-2.06	0.708	0.50	0.26-0.96	0.036
Exposure to cephalosporins						
No						
Yes	3.38	2.46-4.64	<0.001	1.63	1.11-2.38	0.012
Exposure to fluoroquinolones						
No						
Yes	2.26	1.69-3.03	<0.001	1.50	1.06-2.12	0.020
Exposure to glycopeptides						
No						
Yes	3.45	2.50-4.77	<0.001	1.54	1.06-2.24	0.022
Exposure to other antimicrobials						
No						
Yes	6.20	4.10-9.35	<0.001	4.26	2.69-6.76	<0.001
Compliance with hand hygiene at respective site during this hospitalisation[#]						
>75%						
≤75%	1.56	1.19-2.03	0.001	1.12	0.76-1.64	0.582

VRE: vancomycin-resistant enterococci*;* NCP: no contact precautions; SCP: single room contact precautions; SHR: subdistribution hazard ratio; CI: confidence interval
*SHR and 95% CI for SHR obtained from the Fine-Gray model

Table S12b: Variation with separated sites (hospitalisation level)

	Univariate analysis			Multivariate analysis:		
**Variable**	**SHR***	**95% CI**	**p value**	**SHR***	**95% CI**	**p value**
Site						
SCP2						
SCP1	1.76	1.14-2.70	0.010	1.22	0.53-2.84	0.642
NCP1	2.73	1.85-4.01	<0.001	2.77	1.81-4.26	<0.001
NCP2	1.83	1.28-2.61	0.001	0.71	0.28-1.82	0.479
Age						
≤40 years						
41-60 years	1.35	0.87-2.11	0.182	1.40	0.90-2.20	0.137
>60 years	1.15	0.75-1.75	0.526	1.27	0.82-1.95	0.284
Sex						
Female						
Male	1.13	0.86-1.48	0.398	1.14	0.87-1.51	0.345
Underlying disease categorised						
Other						
Acute leukaemia	4.93	3.01-8.05	<0.001	2.09	1.24-3.54	0.006
Lymphoma	1.97	1.20-3.24	0.007	1.05	0.63-1.74	0.851
Solid tumor	1.27	0.74-2.19	0.387	1.16	0.67-2.02	0.597
Exposure to antimicrobials active against VRE (lipopetides and oxazolidinones)						
No						
Yes	1.12	0.61-2.06	0.708	0.57	0.30-1.11	0.099
Exposure to cephalosporins						
No						
Yes	3.38	2.46-4.64	<0.001	1.67	1.11-2.49	0.013
Exposure to fluoroquinolones						
No						
Yes	2.26	1.69-3.03	<0.001	1.46	1.01-2.10	0.043
Exposure to glycopeptides						
No						
Yes	3.45	2.50-4.77	<0.001	1.37	0.92-2.04	0.117
Exposure to other antimicrobials						
No						
Yes	6.20	4.10-9.35	<0.001	4.57	2.75-7.60	<0.001
Compliance with hand hygiene at respective site during this hospitalisation[#]						
>75%						
≤75%	1.56	1.19-2.03	0.001	2.63	1.13-6.13	0.025

VRE: vancomycin-resistant enterococci; NCP: no contact precautions; SCP: single room contact precautions; SHR: subdistribution hazard ratio; CI: confidence interval
*SHR and 95% CI for SHR obtained from the Fine-Gray model

Table S12c: Variation with length of exposure to any antimicrobial class in days (hospitalisation level)

	**Univariate analysis**			**Multivariate analysis:**		
**Variable**	**SHR***	**95% CI**	**p value**	**SHR***	**95% CI**	**p value**
Site group						
SCP						
NCP	1.73	1.32-2.28	<0.001	1.59	1.09-2.33	0.017
Age						
≤40 years						
41-60 years	1.35	0.87-2.11	0.182	1.47	0.94-2.30	0.091
>60 years	1.15	0.75-1.75	0.526	1.28	0.84-1.97	0.256
Sex						
Female						
Male	1.13	0.86-1.48	0.398	1.17	0.89-1.55	0.263
Underlying disease categorised						
Other						
Acute leukaemia	4.93	3.01-8.05	<0.001	4.09	2.46-6.79	<0.001
Lymphoma	1.97	1.20-3.24	0.007	1.57	0.95-2.61	0.079
Solid tumor	1.27	0.74-2.19	0.387	1.13	0.65-1.96	0.658
Length of exposure to any antimicrobial in days**	1.01	1.006-1.014	<0.001	1.01	1.001-1.009	0.008
Compliance with hand hygiene at respective site during this hospitalisation[#]						
>75%						
≤75%	1.56	1.19-2.03	0.001	1.24	0.86-1.79	0.251

VRE: vancomycin-resistant enterococci; NCP: no contact precautions; SCP: single room contact precautions; SHR: subdistribution hazard ratio; CI: confidence interval
*SHR and 95% CI for SHR obtained from the Fine-Gray model
**Days of exposure to different antibiotic classes were summed up irrespective if given in parallel or sequentially.

Table S12d: Variation with length of exposure to any antimicrobial class in days and separate sites (hospitalisation level)

	Univariate analysis			Multivariate analysis:		
**Variable**	**SHR***	**95% CI**	**p value**	**SHR***	**95% CI**	**p value**
CENTER Site group						
SCPsite2						
SCPsite1	1.76	1.14-2.70	0.010	0.47	0.20-1.10	0.082
NCPsite1	2.73	1.85-4.01	<0.001	2.57	1.72-3.84	<0.001
NCPsite2	1.83	1.28-2.61	0.001	0.29	0.11-0.75	0.011
Age						
≤40 years						
41-60 years	1.35	0.87-2.11	0.182	1.45	0.92-2.26	0.106
>60 years	1.15	0.75-1.75	0.526	1.25	0.81-1.91	0.313
Sex						
Female						
Male	1.13	0.86-1.48	0.398	1.17	0.89-1.55	0.267
Underlying disease categorised						
Other						
Acute leukaemia	4.93	3.01-8.05	<0.001	3.63	2.18-6.06	<0.001
Lymphoma	1.97	1.20-3.24	0.007	1.45	0.87-2.40	0.150
Solid tumor	1.27	0.74-2.19	0.387	1.04	0.60-1.82	0.887
Length of exposure to any antimicrobial in days**	1.01	1.006-1.014	<0.001	1.01	1.001-1.010	0.011
Compliance with hand hygiene at respective site during this hospitalisation[#]						
>75%						
≤75%	1.56	1.19-2.03	0.001	6.49	2.72-15.48	<0.001

VRE: vancomycin-resistant enterococci; NCP: no contact precautions; SCP: single room contact precautions; SHR: subdistribution hazard ratio; CI: confidence interval

*SHR and 95% CI for SHR obtained from the Fine-Gray model

**Days of exposure to different antibiotic classes were summed up irrespective if given in parallel or sequentially.

Table S12e: Variation with exposure to any antimicrobial irrespective of length (hospitalisation level)

	**Univariate analysis**			**Multivariate analysis:**		
**Variable**	**SHR***	**95% CI**	**p value**	**SHR***	**95% CI**	**p value**
Site group						
SCP						
NCP	1.73	1.32-2.28	<0.001	1.23	0.83-1.82	0.306
Age						
≤40 years						
41-60 years	1.35	0.87-2.11	0.182	1.44	0.92-2.25	0.111
>60 years	1.15	0.75-1.75	0.526	1.24	0.81-1.91	0.318
Sex						
Female						
Male	1.13	0.86-1.48	0.398	1.19	0.90-1.57	0.220
Underlying disease categorised						
Other						
Acute leukaemia	4.93	3.01-8.05	<0.001	3.13	1.90-5.14	<0.001
Lymphoma	1.97	1.20-3.24	0.007	1.22	0.74-2.03	0.438
Solid tumor	1.27	0.74-2.19	0.387	1.35	0.78-2.34	0.286
Exposure to any antimicrobial						
No						
Yes	8.36	5.09-13.75	<0.001	7.09	4.19-12.00	<0.001
Compliance with hand hygiene at respective site during this hospitalisation[#]						
>75%						
≤75%	1.56	1.19-2.03	0.001	1.42	0.97-2.07	0.068

VRE: vancomycin-resistant enterococci; NCP: no contact precautions; SCP: single room contact precautions; SHR: subdistribution hazard ratio; CI: confidence interval

*SHR and 95% CI for SHR obtained from the Fine-Gray model

Table S12f: Variation with categorised length of exposure to any antimicrobial (hospitalisation level)

	**Univariate analysis**			**Multivariate analysis:**		
**Variable**	**SHR***	**95% CI**	**p value**	**SHR***	**95% CI**	**p value**
Site group						
SCP						
NCP	1.73	1.32-2.28	<0.001	1.29	0.87-1.90	0.204
Age						
≤40 years						
41-60 years	1.35	0.87-2.11	0.182	1.41	0.90-2.21	0.131
>60 years	1.15	0.75-1.75	0.526	1.25	0.82-1.92	0.304
Sex						
Female						
Male	1.13	0.86-1.48	0.398	1.18	0.89-1.56	0.244
Underlying disease categorised						
Other						
Acute leukaemia	4.93	3.01-8.05	<0.001	2.68	1.60-4.48	<0.001
Lymphoma	1.97	1.20-3.24	0.007	1.15	0.69-1.91	0.589
Solid tumor	1.27	0.74-2.19	0.387	1.28	0.73-2.22	0.387
Exposure to any antimicrobial**						
None						
≤7 days	5.50	3.21-9.44	<0.001	5.27	3.02-9.20	<0.001
>7 days	10.94	6.60-18.13	<0.001	8.67	5.03-14.95	<0.001
Compliance with hand hygiene at respective site during this hospitalisation#						
>75%						
≤75%	1.56	1.19-2.03	0.001	1.37	0.95-1.99	0.096

VRE: vancomycin-resistant enterococci; NCP: no contact precautions; SCP: single room contact precautions; SHR: subdistribution hazard ratio; CI: confidence interval
*SHR and 95% CI for SHR obtained from the Fine-Gray model
**Days of exposure to different antibiotic classes were summed up irrespective if given in parallel or sequentially.

Table S12g: Variation with length of exposure to antimicrobials active or not active against VRE in days (hospitalisation level)

	Univariate analysis			Multivariate analysis:		
**Variable**	**SHR***	**95% CI**	**p value**	**SHR***	**95% CI**	**p value**
Site group						
SCP						
NCP	1.73	1.32-2.28	<0.001	1.55	1.06-2.25	0.022
Age						
≤40 years						
41-60 years	1.35	0.87-2.11	0.182	1.47	0.93-2.31	0.096
>60 years	1.15	0.75-1.75	0.526	1.34	0.86-2.07	0.191
Sex						
Female						
Male	1.13	0.86-1.48	0.398	1.17	0.89-1.55	0.269
Underlying disease categorised						
Other						
Acute leukaemia	4.93	3.01-8.05	<0.001	3.96	2.39-6.59	<0.001
Lymphoma	1.97	1.20-3.24	0.007	1.60	0.97-2.66	0.068
Solid tumor	1.27	0.74-2.19	0.387	1.15	0.67-2.00	0.611
Length of exposure to antimicrobials active against VRE in days (lipopeptides and oxazolidinones)**	0.98	0.93-1.03	0.440	0.92	0.87-0.98	0.006
Length of exposure to -antimicrobials not active against VRE in days**	1.01	1.01-1.02	<0.001	1.01	1.01-1.02	<0.001
Compliance with hand hygiene at respective site during this hospitalisation#						
>75%						
≤75%	1.56	1.19-2.03	0.001	1.16	0.81-1.66	0.430

VRE: vancomycin-resistant enterococci; NCP: no contact precautions; SCP: single room contact precautions; SHR: subdistribution hazard ratio; CI: confidence interval

*SHR and 95% CI for SHR obtained from the Fine-Gray model

**Days of exposure to different antibiotic classes were summed up irrespective if given in parallel or sequentially.

Table S12h: Variation with exposure to antimicrobials active or not active against VRE irrespective of length (hospitalisation level)

	Univariate analysis			Multivariate analysis:		
**Variable**	**SHR***	**95% CI**	**p value**	**SHR***	**95% CI**	**p value**
Site group						
SCP						
NCP	1.73	1.32-2.28	<0.001	1.20	0.81-1.77	0.372
Age						
≤40 years						
41-60 years	1.35	0.87-2.11	0.182	1.45	0.92-2.27	0.107
>60 years	1.15	0.75-1.75	0.526	1.25	0.81-1.91	0.316
Sex						
Female						
Male	1.13	0.86-1.48	0.398	1.19	0.91-1.57	0.210
Underlying disease categorised						
Other						
Acute leukaemia	4.93	3.01-8.05	<0.001	3.30	2.00-5.46	<0.001
Lymphoma	1.97	1.20-3.24	0.007	1.25	0.75-2.08	0.384
Solid tumor	1.27	0.74-2.19	0.387	1.36	0.79-2.36	0.272
Exposure to antimicrobials active against VRE (lipopetides and oxazolidinones)						
No						
Yes	1.12	0.61-2.06	0.708	0.61	0.32-1.16	0.131
Exposure to Non-antimicrobials not active against VRE						
No						
Yes	8.36	5.09-13.75	<0.001	7.28	4.31-12.31	<0.001
Compliance with hand hygiene at respective site during this hospitalisation#						
>75%						
≤75%	1.56	1.19-2.03	0.001	1.36	0.94-1.97	0.108

VRE: vancomycin-resistant enterococci; NCP: no contact precautions; SCP: single room contact precautions; SHR: subdistribution hazard ratio; CI: confidence interval

*SHR and 95% CI for SHR obtained from the Fine-Gray model

Table S12i: Variation with categorised length of antimicrobials active or not active against VRE (hospitalisation level)

	Univariate analysis			Multivariate analysis:		
**Variable**	**SHR***	**95% CI**	**p value**	**SHR***	**95% CI**	**p value**
Site group						
SCP						
NCP	1.73	1.32-2.28	<0.001	1.23	0.84-1.81	0.290
Age						
≤40 years						
41-60 years	1.35	0.87-2.11	0.182	1.43	0.91-2.24	0.122
>60 years	1.15	0.75-1.75	0.526	1.25	0.82-1.93	0.301
Sex						
Female						
Male	1.13	0.86-1.48	0.398	1.19	0.90-1.57	0.217
Underlying disease categorised						
Other						
Acute leukaemia	4.93	3.01-8.05	<0.001	2.86	1.71-4.80	<0.001
Lymphoma	1.97	1.20-3.24	0.007	1.19	0.71-1.98	0.504
Solid tumor	1.27	0.74-2.19	0.387	1.29	0.74-2.23	0.372
Length of exposure to antimicrobials active against VRE in categories (lipopetides and oxazolidinones)**						
None						
≤7 days	1.52	0.72-3.23	0.275	0.80	0.37-1.74	0.568
>7 days	0.77	0.29-2.07	0.605	0.32	0.12-0.89	0.029
Length of exposure to Non-antimicrobials not active against VRE antimicrobials in categories**						
None						
≤7 days	5.51	3.21-9.45	<0.001	5.28	3.03-9.20	<0.001
>7 days	11.00	6.64-18.24	<0.001	9.19	5.34-15.81	<0.001
Compliance with hand hygiene at respective site during this hospitalisation#						
>75%						
≤75%	1.56	1.19-2.03	0.001	1.29	0.89-1.86	0.180

VRE: vancomycin-resistant enterococci; NCP: no contact precautions; SCP: single room contact precautions;
SHR: subdistribution hazard ratio; CI: confidence interval
*SHR and 95% CI for SHR obtained from the Fine-Gray model
**Days of exposure to different antibiotic classes were summed up irrespective if given in parallel or sequentially.

Table S12j: Variation with length of exposure to antimicrobial class in days (hospitalisation level)

	Univariate analysis			Multivariate analysis:		
**Variable**	**SHR***	**95% CI**	**p value**	**SHR***	**95% CI**	**p value**
Site group						
SCP						
NCP	1.73	1.32-2.28	<0.001	1.80	1.23-2.61	0.002
Age						
≤40 years						
41-60 years	1.35	0.87-2.11	0.182	1.48	0.95-2.32	0.084
>60 years	1.15	0.75-1.75	0.526	1.30	0.85-2.00	0.229
Sex						
Female						
Male	1.13	0.86-1.48	0.398	1.22	0.92-1.60	0.163
Underlying disease categorised						
Other						
Acute leukaemia	4.93	3.01-8.05	<0.001	3.78	2.25-6.36	<0.001
Lymphoma	1.97	1.20-3.24	0.007	1.57	0.95-2.61	0.080
Solid tumor	1.27	0.74-2.19	0.387	1.14	0.66-1.98	0.631
Length of exposure to antimicrobials active against VRE in days (lipopetides and oxazolidinones) **	0.98	0.93-1.03	0.440	0.86	0.79-0.94	0.001
Length of exposure to cephalosporins in days**	1.04	1.02-1.06	<0.001	1.05	1.01-1.08	0.014
Length of exposure to fluoroquinolones in days**	1.05	1.03-1.07	<0.001	1.07	1.04-1.10	<0.001
Length of exposure to glycopeptides in days**	1.03	1.01-1.06	0.001	0.97	0.94-1.00	0.080
Length of other antimicrobials in days**	1.02	1.01-1.03	<0.001	1.01	1.00-1.02	0.133
Compliance with hand hygiene at respective site during this hospitalisation#						
>75%						
≤75%	1.56	1.19-2.03	0.001	1.07	0.75-1.53	0.700

VRE: vancomycin-resistant enterococci; NCP: no contact precautions; SCP: single room contact precautions; SHR: subdistribution hazard ratio; CI: confidence interval

*SHR and 95% CI for SHR obtained from the Fine-Gray model

**Days of exposure to different antibiotic classes were summed up irrespective if given in parallel or sequentially.

Table S12k: Variation with categorised length of antimicrobial classes (hospitalisation level)

	Univariate analysis			Multivariate analysis:		
**Variable**	**SHR***	**95% CI**	**p value**	**SHR***	**95% CI**	**p value**
Site group						
SCP						
NCP	1.73	1.32-2.28	<0.001	1.67	1.12-2.50	0.012
Age						
≤40 years						
41-60 years	1.35	0.87-2.11	0.182	1.40	0.89-2.20	0.142
>60 years	1.15	0.75-1.75	0.526	1.30	0.85-2.00	0.228
Sex						
Female						
Male	1.13	0.86-1.48	0.398	1.21	0.92-1.60	0.171
Underlying disease categorised						
Other						
Acute leukaemia	4.93	3.01-8.05	<0.001	2.44	1.43-4.15	0.001
Lymphoma	1.97	1.20-3.24	0.007	1.13	0.68-1.87	0.649
Solid tumor	1.27	0.74-2.19	0.387	1.24	0.71-2.15	0.446
Length of exposure to antimicrobials active against VRE in categories (lipopetides and oxazolidinones) **						
None						
≤7 days	1.52	0.72-3.23	0.275	0.81	0.37-1.77	0.594
>7 days	0.77	0.29-2.07	0.605	0.26	0.09-0.75	0.012
Length of exposure to cephalosporins in categories**						
None						
≤7 days	3.48	2.46-4.93	<0.001	1.82	1.21-2.75	0.004
>7 days	3.03	1.61-5.72	0.001	1.45	0.70-3.02	0.318
Length of exposure to fluoroquinolones in categories**						
None						
≤7 days	1.59	1.07-2.35	0.022	1.21	0.80-1.84	0.369
>7 days	3.50	2.43-5.04	<0.001	2.26	1.39-3.68	0.001
Length of exposure to glycopeptides in categories**						
None						
≤7 days	3.64	2.50-5.29	<0.001	1.80	1.17-2.76	0.008
>7 days	3.04	1.76-5.28	<0.001	1.02	0.55-1.90	0.950
Length of other antimicrobials**						
None						
≤7 days	5.97	3.88-9.21	<0.001	4.50	2.82-7.18	<0.001
>7 days	6.49	4.18-10.08	<0.001	3.68	2.19-6.19	<0.001
Compliance with hand hygiene at respective site during this hospitalisation#						
>75%						
≤75%	1.56	1.19-2.03	0.001	1.06	0.72-1.55	0.774

VRE: vancomycin-resistant enterococci; NCP: no contact precautions; SCP: single room contact precautions; SHR: subdistribution hazard ratio; CI: confidence interval

*SHR and 95% CI for SHR obtained from the Fine-Gray model

**Days of exposure to different antibiotic classes were summed up irrespective if given in parallel or sequentially.

Table S13: Numbers of hospitalisations with completed surveillance screenings

	**NCP (%) N=2435**	**SCP (%) N=3023**
Admission screening completed within 72 hours after admission	2234 (91.7)	2694 (89.1)
Weekly screening completed for whole hospitalisation	2060 (84.6)	2782 (92.0)
Discharge screening completed within 72 hours before discharge	1865 (76.6)	2484 (82.2)
Admission screening and weekly screening completed for whole hospitalisation	2010 (82.5)	2628 (86.9)
Admission screening completed plus screening within 7 days of discharge	2159 (88.7)	2652 (87.7)
Admission screening, weekly screening and discharge screening within 72 hours before discharge	1719 (70.6)	2299 (76.1)
At least two screening samples completed during hospitalisation*	1528 (62.8)	1858 (61.5)

NCP: no contact precautions; SCP: single room contact precautions
§ Fisher's exact test
*Of note, during short hospitalisations of ≤5 days, taking just one sample still complied with the sample protocol.

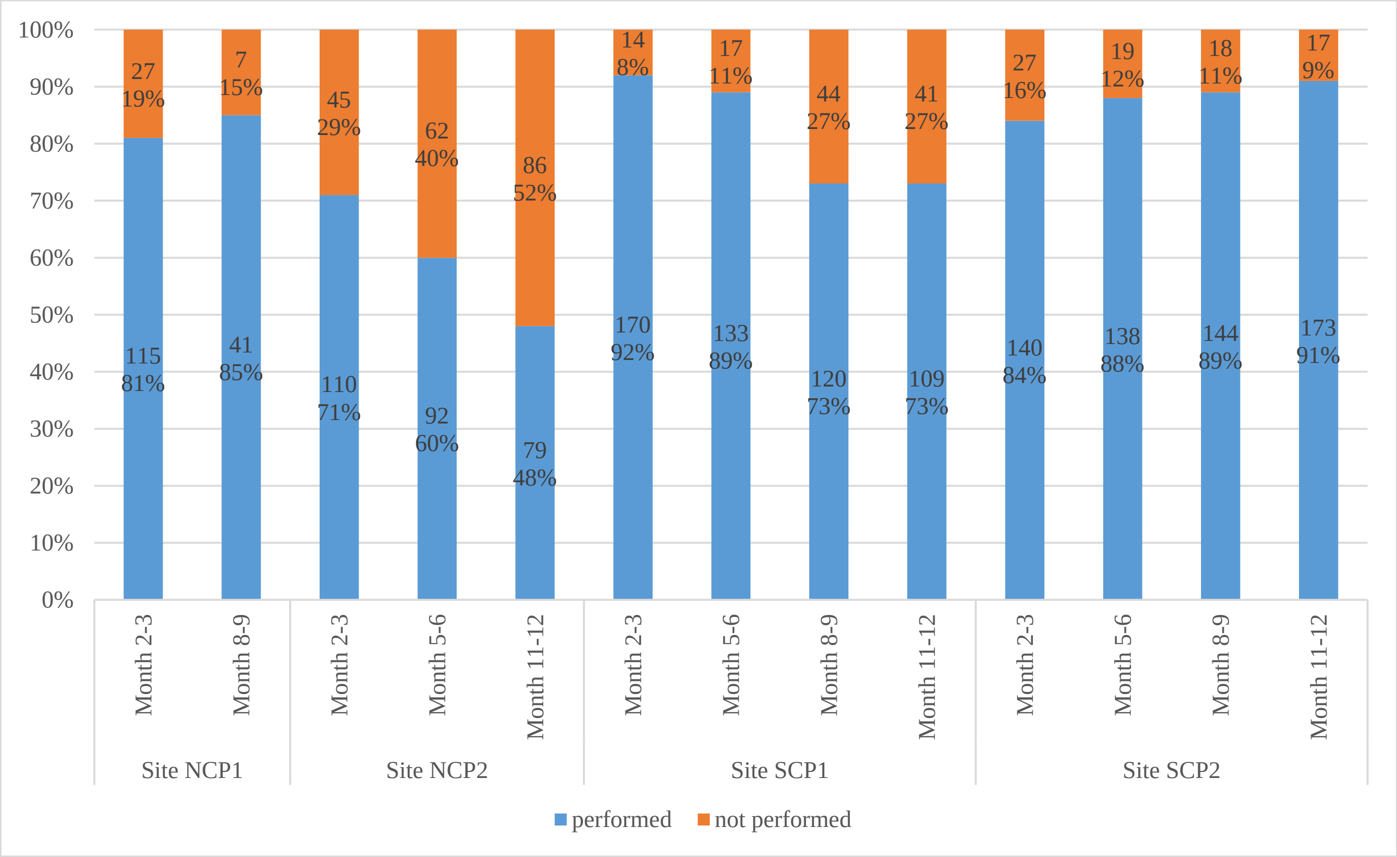

Figure S4: Hand hygiene compliance as determined during observations over the study period
Numbers are counts of correctly or not correctly performed hand hygiene indications. During each observation, each site was asked to observe approximately 150 indications for hand hygiene according to the WHO 5 moments for hand hygiene. NCP sites did not complete all four observations.
NCP: no single room contact precautions; SCP: single room contact precautions

Table S14: Defined daily doses of antimicrobial classes per 100 patient days of participating study sites and per quarter

	Site NCP1 DDD/100pd				Site NCP2 DDD/100pd				Site SCP1 DDD/100pd				Site SCP2 DDD/100pd			
	**Q1**	**Q2**	**Q3**	**Q4**	**Q1**	**Q2**	**Q3**	**Q4**	**Q1**	**Q2**	**Q3**	**Q4**	**Q1**	**Q2**	**Q3**	**Q4**
3GC/4GC	1.79	4.04	1.58	1.39	6.90	5.16	8.16	6.18	15.92	22.0	23.23	15.18	7.77	5.49	9.04	6.45
BLBLI	21.76	17.64	19.41	17.71	14.57	13.67	15.03	12.16	17.12	15.99	12.90	14.56	22.43	28.58	28.90	27.30
Carbapenems	20.46	16.80	20.77	25.31	13.55	8.92	13.07	13.50	31.81	20.61	21.26	28.34	26.53	28.07	26.03	21.83
Fluoroquinolones	12.86	10.53	8.17	13.83	6.16	5.78	7.32	7.73	27.24	27.06	34.50	40.74	31.44	35.63	29.95	28.02
Macrolides	5.91	6.34	7.00	6.09	2.15	4.77	5.19	6.49	6.36	6.09	2.37	7.81	7.83	13.57	7.56	8.37
Vancomycin*	10.31	7.28	3.70	4.42	3.45	4.39	4.61	2.80	13.77	14.46	18.09	18.69	6.74	3.69	2.56	3.27
Metronidazol	4.02	1.50	0.23	2.08	0.69	0.07	0.10	0.32	1.73	3.48	2.02	3.38	3.57	3.37	1.82	2.83
Linezolid/ Daptomycin	7.43	8.28	15.84	11.29	1.00	0.75	1.13	0.34	6.64	5.10	6.15	9.41	10.25	14.55	14.69	8.67

NCP: no single room contact precautions; SCP: single room contact precautions; DDD: defined daily doses;
pd: patient days; Q: quarter; 3GC/4GC: third and fourth generation cephalosporin; BLBLI: Beta lactam beta-lactamase inhibitor combination
* Nearly no other glycopeptides used

Table S15: Average quarterly defined daily doses per 100 patient days of participating sites

	Site NCP1 DDD/100pd	Site NCP2 DDD/100pd	Site SCP1 DDD/100pd	Site SCP2 DDD/100pd
	**Average/quarter**	**Average/quarter**	**Average/quarter**	**Average/quarter**
3GC/4GC	2.20	6.60	19.08	7.19
BLBLI	19.13	13.86	15.14	26.80
Carbapenems	20.84	12.26	25.51	25.62
Fluoroquinolones	11.35	6.75	32.39	31.26
Macrolides	6.34	4.65	5.66	9.33
Vancomycin*	6.43	3.81	16.50	4.07
Metronidazol	1.96	0.295	2.65	2.90
Linezolid/Daptomycin	10.71	0.81	6.82	12.05
**Total of listed groups**	78.96	49.035	123.75	119.22

NCP: No single room contact precautions; SCP: single room contact precautions
DDD: defined daily doses; pd: patient days; 3GC/4GC: third and fourth generation cephalosporin; BLBLI: Beta lactam beta-lactamase inhibitor combination;
* Nearly no other glycopeptides used

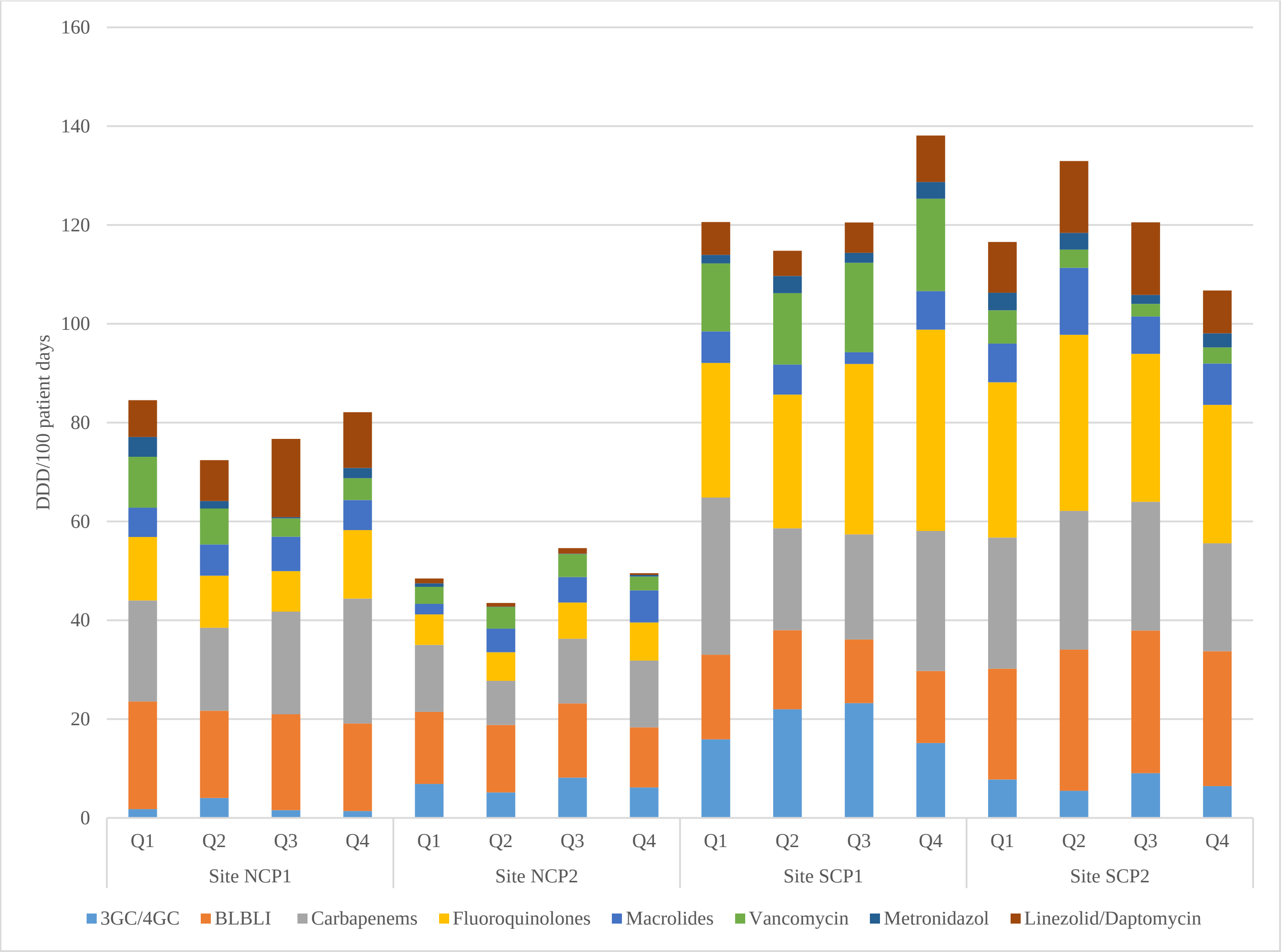

Figure S5: Antimicrobial consumption in defined daily doses per 100 patient days
NCP: No single room contact precautions; SCP: single room contact precautions
DDD: defined daily doses; Q: quarter; 3GC/4GC: third and fourth generation cephalosporin; BLBLI: Beta lactam beta-lactamase inhibitor combination;

References

1. Liss BJ, Vehreschild JJ, Cornely OA, Hallek M, Fatkenheuer G, Wisplinghoff H, et al. Intestinal colonisation and blood stream infections due to vancomycin-resistant enterococci (VRE) and extended-spectrum beta-lactamase-producing Enterobacteriaceae (ESBLE) in patients with haematological and oncological malignancies. Infection. 2012;40(6):613-9.

2. Fine JP, Gray RJ. A Proportional Hazards Model for the Subdistribution of a Competing Risk. Journal of the American Statistical Association. 1999;94(446):496-509.

3. Papadimitriou-Olivgeris M, Drougka E, Fligou F, Kolonitsiou F, Liakopoulos A, Dodou V, et al. Risk factors for enterococcal infection and colonization by vancomycin-resistant enterococci in critically ill patients. Infection. 2014;42(6):1013-22.

4. Almyroudis NG, Lesse AJ, Hahn T, Samonis G, Hazamy PA, Wongkittiroch K, et al. Molecular epidemiology and risk factors for colonization by vancomycin-resistant Enterococcus in patients with hematologic malignancies. Infection control and hospital epidemiology. 2011;32(5):490-6.

5. Zhou Q, Moore C, Eden S, Tong A, McGeer A. Factors associated with acquisition of vancomycin-resistant enterococci (VRE) in roommate contacts of patients colonized or infected with VRE in a tertiary care hospital. Infection control and hospital epidemiology. 2008;29(5):398-403.
